# Cell Wall Metabolism in Response to Abiotic Stress

**DOI:** 10.3390/plants4010112

**Published:** 2015-02-16

**Authors:** Hyacinthe Le Gall, Florian Philippe, Jean-Marc Domon, Françoise Gillet, Jérôme Pelloux, Catherine Rayon

**Affiliations:** EA3900-BIOPI, Biologie des Plantes et Innovation, Université de Picardie Jules Verne, 80039 Amiens, France; E-Mails: hyacinthe.le.gall@u-picardie.fr (H.L.G.); florian.philippe@u-picardie.fr (F.P.); jean-marc.domon@u-picardie.fr (J.-M.D.); francoise.gillet@u-picardie.fr (F.G.); jerome.pelloux@u-picardie.fr (J.P.)

**Keywords:** plant cell wall, abiotic stress, water deficit, temperature, salt, flooding, heavy metals, light, air pollutants

## Abstract

This review focuses on the responses of the plant cell wall to several abiotic stresses including drought, flooding, heat, cold, salt, heavy metals, light, and air pollutants. The effects of stress on cell wall metabolism are discussed at the physiological (morphogenic), transcriptomic, proteomic and biochemical levels. The analysis of a large set of data shows that the plant response is highly complex. The overall effects of most abiotic stress are often dependent on the plant species, the genotype, the age of the plant, the timing of the stress application, and the intensity of this stress. This shows the difficulty of identifying a common pattern of stress response in cell wall architecture that could enable adaptation and/or resistance to abiotic stress. However, in most cases, two main mechanisms can be highlighted: (i) an increased level in xyloglucan endotransglucosylase/hydrolase (XTH) and expansin proteins, associated with an increase in the degree of rhamnogalacturonan I branching that maintains cell wall plasticity and (ii) an increased cell wall thickening by reinforcement of the secondary wall with hemicellulose and lignin deposition. Taken together, these results show the need to undertake large-scale analyses, using multidisciplinary approaches, to unravel the consequences of stress on the cell wall. This will help identify the key components that could be targeted to improve biomass production under stress conditions.

## 1. Introduction

During their entire life cycle, plants are exposed to various environmental stresses. Two major categories can be distinguished: abiotic stress, which encompasses a variety of unfavorable environmental conditions, such as drought, submergence, salinity, heavy metal contamination and nutrient deficiency, and biotic stress caused by infectious living organisms, such as bacteria, viruses, fungi, or nematodes. Both types of stress negatively affect the productivity and survival of plants [1]. Abiotic stress reduces agricultural yield, so that novel crop genotypes adapted to environmental stress need to be developed [2,3,4]. Restriction of plant growth by retarding cell extension is often the earliest visible effect of stress. Plants have developed a range of morphological, physiological and biochemical mechanisms that enable them to avoid and/or tolerate stress factors and survive [5]. Reactive oxygen species (ROS) homeostasis is changed in response to stress; they are regarded as molecules causing damage to cells at high concentration as well as ubiquitous signaling molecules at low concentration, thus participating in recognizing and responding to stress factors [6]. Transduction of the signal modifies gene and protein expression levels leading to physiological responses. ROS are produced in most plant subcellular locations including the plasma membrane, mitochondria, nucleus, chloroplast and cell wall [7].

The plant cell wall determines cell size and shape through the mechanical control of cell expansion, which governs tissue and organ morphology. It can be considered a protective barrier and is a complex structure composed of cellulose microfibrils and non-cellulosic neutral polysaccharides embedded in a physiologically active pectin matrix, cross-linked with structural proteins and, depending on the tissue/organ, with lignin [8,9]. Plant cell walls are divided into a primary wall, which is initiated during cell division and deposited during cell elongation, and a secondary wall, which is deposited within the primary wall of specific cell types with specialized functions. In parallel, depending on the presence/absence of some particular polysaccharides, type I and type II cell walls can be distinguished. Type I primary walls are a framework of cellulose microfibrils, mostly cross-linked with xyloglucans (XG) and embedded in a complex matrix of pectic polysaccharides [8,9]. Unlike type I, type II primary cell walls contain small amounts of pectic polysaccharides and xyloglucans, whereas glucuronoarabinoxylans (GAX) and mixed-linkage (1→3),(1→4)-β-D-glucan (β-glucan) are the main matrix polymers that interlace cellulose microfibrils [10]. Furthermore, ferulic and *p*-coumaric acid arabinosyl esters can cross-link GAX in type II primary walls, as well as in the secondary wall, to stiffen the matrix [11,12]. Cell wall-related proteins are believed to play a central role in modulating cell wall extensibility, which mediates cell enlargement and expansion. These proteins include xyloglucan endo-β-transglucosylases/hydrolases (XET/XTHs; GH16), endo-1,4-β-D-glucanase (EGase, GH9), and expansins (EXPA, EXPB, EXPLA, EXPLB; GH45) [13,14]. Other cell wall-modifying enzymes play a major role in controlling cell wall plasticity/rheology. These include pectin-modifying enzymes such as pectin methylesterase (PME; CE8), polygalacturonase (PG; GH28), pectin/pectate lyase-like (PLL; PL1) and pectin acetylesterase (PAE; CE13) [15].

The massive deposition of cellulose and hemicelluloses inside primary walls gives secondary walls their characteristic thickness [16]. During secondary cell wall formation, monolignols, precursors of lignin, are secreted into the cell wall space and randomly cross-linked through oxidative polymerization [17]. The cross-linking is dependent on the availability of ROS generated by laccases and cell wall peroxidases (PRX). This process reinforces the strength and rigidity of the cell walls and can be a key component of the plant response to environmental factors [17,18,19]. Cell wall architecture is important in plant resistance to abiotic stress and essential in stress sensing and signal transduction [20]. A summary of state of the art of recent reviews is presented in Table 1.

**Table 1:** List of references of recent reviews on the effects of abiotic stress on cell wall metabolism. References to the papers are given in brackets.

Reference	Stress	Cell Wall	Data Studied
[21]	C, Fl, L, Wd	Primary cell wall	Transcriptomic and proteomic
[22]	Wd	Primary and secondary cell wall	Physiological, proteomic and cell wall composition
[23]	Ap, HM, L, Wd	Secondary cell wall	Transcriptomic, proteomic and metabolomic
[24]	Wd, S, C, Ap, HM, L	Secondary cell wall	Transcriptomic, proteomic and cell wall composition
[25]	S	Primary cell wall	Transcriptomic and proteomic
[26]	S	Primary and secondary cell wall	Proteomic
[27]	HM	Primary cell wall	Transcriptomic and proteomic
[28]	HM	Primary and secondary cell wall	Transcriptomic and proteomic

Ap: Air pollutants; C: Cold; Fl: Flooding; HM: Heavy Metal; L: Light; S: Salt stress; Wd: Water deficit.

The present review summarizes the most recent insights into the effects of eight abiotic stress factors (drought, flooding, heat, cold, salinity, metals, light irradiance, and air pollutants) on primary and secondary cell wall metabolism. Although callose synthesis and deposition have been observed in response to described abiotic stress factors, this polymer is usually not present in the walls of most cell types and is thus beyond the scope of the present review (For reviews: [29,30]). Although the cuticle composed of cutin and waxes, interacts with cell wall, this review will not extend on the effect of abiotic stress on plant cuticle, and will only focus on cell wall components involved in cell wall architecture [31].

## 2. Water Deficit

Plant growth requires concerted water uptake and irreversible cell wall expansion to enlarge cells [32]. Drought or dehydration results in water deficit stress. Water deficit is defined as an imbalance between soil water availability and evaporative demand [33]. It is one of the major environmental stresses that reduces plant growth and productivity [34]. It is generally agreed that drought stress generates physiological changes in higher plants, including loss of turgor, osmotic adjustment, and reduced leaf water potential (for reviews: [35,36]). Turgor pressure is a critical factor in regulating cell growth. It is the physical force needed to drive cell enlargement, which notably depends upon the extensibility of the cell wall [37,38]. A low turgor pressure caused by water stress leads to a reduction or cessation of growth by decreasing cell extensibility and cell expansion [39]. Plants exposed to water deficit display morphological changes that are the result of plant cell wall modifications. In Black spruce(*Picea mariana*) saplings, drought stress induced a decrease in cell wall thickness, reflecting a lower carbon allocation to cell wall formation and preventing the adaptation of the hydraulic system to drought [40]. Cell expansion can only occur when turgor pressure is greater than the cell wall yield threshold [33,35]. In winter triticale, water deficit induced leaf rolling, which was correlated with a higher level of cell wall-bound phenolics [41]. This adaptive mechanism can limit water loss by restricting the leaf transpiration surface [42,43].

In response to stress, an increase in cell wall elasticity (CWE) can contribute to the maintenance of cell turgor or symplast volume [44,45,46]. CWE can be correlated with plant drought tolerance [46,47], as shown by a study performed on six cultivars of common bean, which highlighted that the cultivars most resistant to drought showed a strong decrease in their elasticity modulus (ε) in association with a higher CWE; this ultimately enabled an osmotic adjustment [47]. In contrast, the highly drought-sensitive cultivars did not show any significant change in their ε and CWE. These variations in ε and CWE in response to water stress presumably reflect differences in wall structure. However, these changes could depend on the species considered, as in *Ziziphus jujuba* and smooth cordgrass (*Spartina alterniflora*) a large increase in ε was observed, thus indicating that rigid cell walls in leaves may be necessary to maintain cell/tissue integrity during rehydration following a period of stress [37,48,49].

A number of studies report that cellulose biosynthesis can be altered in response to water deficit, as shown by the decreased level of cellulose content in several different species including Arabidopsis, tobacco suspension cells, grape leaves and wheat roots [50,51,52,53]. However, in other studies, an increased level of UDP-Glc in the expression of *SuSy* (sucrose synthase) and UDP-glucose pyrophosphorylase (*UGPase*) encoding genes was observed in cotton under drought stress, suggesting a potentially higher cellulose biosynthesis [54]. Increased cellulose synthesis could be a means by which cell wall integrity and cell turgor pressure are maintained, thus allowing continuous cell growth under low water potential [55]. Xyloglycan biosynthesis seems to follow a similar trend in the elongation zone of rice roots under water deficit [56]. In that study, *XTH* and xylose isomerase genes encoding two xyloglucan-modifying enzymes were up-regulated in the early stages following stress application, indicating a role of xyloglucan in the maintenance of root growth [56]. Similarly, the overexpression of an *XTH* gene from pepper in transgenic plants confirmed the role of XTH in enabling better drought tolerance [57,58]. This appears to be a common feature of distinct species, as transgenic Arabidopsis plants overexpressing a hot pepper (*Capsicum annuum*, cv. Pukang) *XTH* (*CaXTH3*) exhibited abnormal leaf morphology resulting in a severely wrinkled leaf shape, an increase in the number of small-sized cells in the leaf mesophyll cells and, ultimately, an increased tolerance to severe water deficit. This suggests that CaXTH3 may be involved in cell wall remodeling to strengthen the wall layers, and hence it could participate in the protection of mesophyll cells against water deficit [57]. In another study performed on transgenic tomato plants overexpressing *CaXTH3*, similar results were shown, highlighting the key role of XTH in water-deficit tolerance [58]. This can be further illustrated by a proteomic analysis in maize, which showed that XTH proteins were differentially regulated upon drought stress exposure [59]. In tomato, a drought responsive transcription factor ASR1 (Abscisic Stress Ripening) was identified as interacting with *Solyc08g082650.2.1*, which was annotated as a cellulose synthase-like (*CSL-G*) involved in hemicellulose biosynthesis [55]. Expansin, another cell wall protein involved in cell wall extension, appears to be strongly regulated by water stress deficiency [60]. In maize roots, expansin activity increased by 4-fold (apical region) and by 2-fold (basal region) under low water potential, which could be related to the higher expression of expansin encoding genes in the root apical zone, corresponding to maintaining elongation [61,62]. However, surprisingly, in the basal region of maize roots, the cell wall underwent a stiffening reaction rather than extension in response to expansin, thus indicating that loss of susceptibility to expansin was associated with growth arrest in this area [61,63]. In soybean, the expression of *GmEXPB2*, a gene that encodes a β-expansin (*EXPB*), was induced by mild water deficiency [64]. This gene, which is intrinsically involved in the response of the root system architecture to some abiotic stresses, including water deficiency, could be an interesting target for improving crop production under these conditions. In a similar way, the overexpression of the wheat β-expansin *EXPB23* under the control of a stress inducible promoter (RD29) in transgenic tobacco plant conferred a higher tolerance to drought stress [65]. The potential key role of expansin in drought tolerance can be further illustrated by studies showing that, in rose (*Rosa hybrida*, cv. Samantha), the virus-induced gene silencing of *RhNAC2*, a transcription factor, or *RhEXPA4*, decreased dehydration tolerance during cell expansion of rose petals [66]. The authors suggested that *RhEXPA4* expression might be regulated by *RhNAC2*. Overexpression of *RhEXPA4* in Arabidopsis transgenic plants conferred a strong phenotype with shorter stems, curly leaves, compact inflorescences and strong drought tolerance, indicating the key role of EXP in response to water deficit in the plant [67]. In wheat coleoptiles, PEG treatment, which can be used to modify osmotic potential and thus induce plant water deficit, limited the increase in the amounts of middle- and low-molecular-weight polysaccharides. In contrast, no effect was shown on high-molecular-weight polysaccharides, such as (1→3),(1→4)-β-D-glucan and arabinoxylan, despite growth arrest [68]. These results demonstrated that the cell wall maintained its ability to extensibility.

In parallel with observations on cellulose and hemicellulose, pectins have been shown to play a key role in modulating cell wall structure in response to drought stress. This fits well with the recognized importance of pectin in controlling plant growth and development (for reviews: [38,69]). In particular, the large amount of homogalacturonan (HG), the most abundant pectic polymer in type I cell walls, combined with a precise regulation of its degree of methylesterification, is likely to be a key element in the control of the stiffness and hydration status of the pectic matrix during drought stress. In addition, it was shown that the amount of side chains of RGI and/or RGII determined the hydration status of the cell wall matrix [70]. The comparison of two wheat cultivars, one tolerant and one sensitive to water stress deficiency, shed new light on the role of this polymer. In the tolerant cultivar, the amount of side chains was indeed increased during water stress, with consequent effects on the viscosity status of the cell wall [52,70]. This latter parameter could be an indicator of drought tolerance [52,70]. In some plants, known as resurrection plants, which can recover completely from a fully-dehydrated state, side chains of pectin are highly enriched in arabinose-rich polymers, including pectin-arabinan, arabinogalactan proteins and arabinoxylans [22,71,72]. The presence of arabinan-rich pectin would prevent water loss during desiccation. In parallel, pectin-degrading enzymes, including PG, can be down-regulated by water stress with consequences on cell wall integrity and cell expansion [53,73]. Alternatively, PG could be involved in controlling cellular water relationships [53,74]. In another study on date palm fruit, the level of methylesterification and *O*-acetylation of xyloglucan was reduced up to 75% and 38% respectively 232 days after pollination, indicating cell wall remodeling of fruits in a water deficit environment [71]. The overexpression of *OsBURP16*, which encodes a putative precursor of PG1β, one of the subunits that regulates PG activity in rice transgenic plants, led to an increase in PG activity and a decrease in pectin content [75,76]. Thus, an increase in the sensitivity of these transgenic rice plants to water stress deficit was shown. In these plants, the intercellular spaces of leaves were larger, suggesting that cell adhesion could be reduced. HG modifications are likely to be an important determinant of stress tolerance as transgenic Arabidopsis, overexpressing a pepper PME inhibitor protein (CaPMEI1), exhibited enhanced tolerance to drought stress during post-germination growth [77].

**Figure 1:**
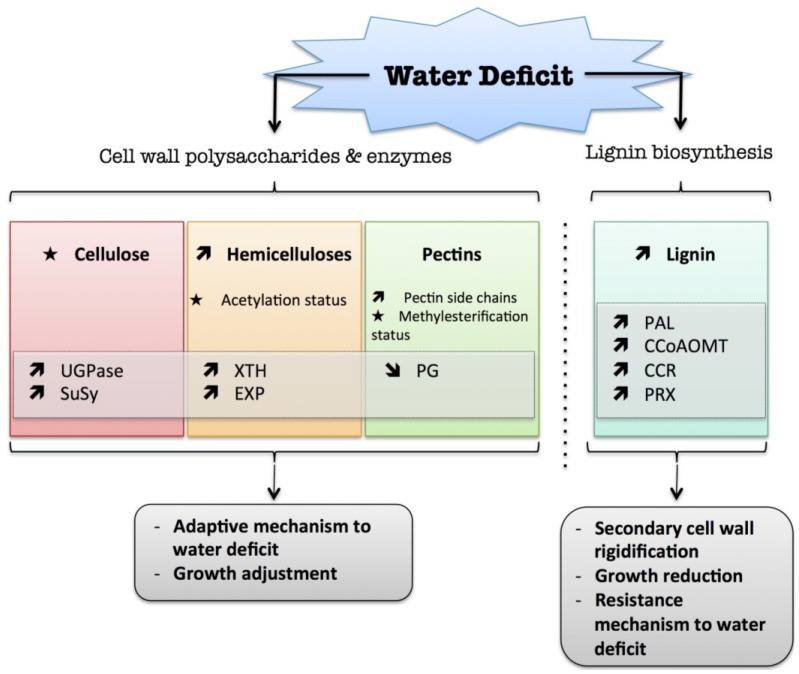
Diagram summarizing the plant cell wall response to water deficit. The schematic presentation is deduced from the results of different studies reported in the review. Arrow (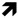) means increased abundance and arrow (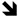) means decreased abundance of the molecules. Star (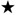) means contrasting data on the gene/protein or the molecule studied according to the literature cited in the review. Cell wall (CW); xyloglucan endo-β-transglucosylases/hydrolases (XET/XTH); expansin (EXP); sucrose synthase (SuSy); UDP-glucose pyrophosphorylase (UGPase); polygalacturonase (PG); pectin methylesterase (PME); phenylalanine ammonia-lyase (PAL); caffeoyl-CoA 3-*O*-methyl-transferase (CCoAOMT); cinnamoyl-CoA reductase (CCR); cell wall peroxidases (PRX).

The secondary cell wall can be strengthened by the incorporation of lignin. Lignification is a complex process involving several different phenolic substrates and enzymes (for a review: [78]). It can occur prematurely to avoid cell wall damage when plants are exposed to a long water stress deficit [23]. Two recent reviews highlighted changes in lignin content and structure following biotic and abiotic stress ([23,24]). Phenylalanine ammonia-lyase (PAL) is a key intermediate at the crossroads of phenolics and lignin synthetic pathways. PAL, which catalyzes the deamination of phenylalanine to trans-cinnamate, was shown to be regulated upon stress exposure. In rice, a proteomic approach showed an increased level of PAL following drought stress [73]. In Grey-maranta (*Ctenanthe setosa*) exposed to long-term drought stress, PAL activity was increased up to 16-fold, with consequent effects on lignin content, ionically wall-bound peroxidase and polyphenol oxidase activities and leaf rolling [79]. The caffeoyl-CoA 3-*O*-methyl-transferase (*CCoAOMT*) encoding gene, which converts caffeoyl-CoA into feruloyl-CoA and thus plays a crucial role in the synthesis of monolignols, was up-regulated in roots of wild water melon subjected to drought [80]. The increase in cinnamoyl-CoA reductase (*CCR*) transcripts in maize roots was associated with a progressive inhibition of wall extensibility and root growth, and may facilitate root acclimation to drying environments [81]. In Eucalyptus, drought stress caused disruption of lignin deposition in leaves and increased the S/G unit ratio [82]. In roots of rice seedlings, the increase in peroxidase activity was correlated with cell wall stiffening, which could be involved in the regulation of root growth reduction caused by water deficit [83]. In white clover leaf, a low water potential enhanced the activation of guaiacol peroxidase and coniferyl alcohol peroxidase, which was closely correlated with an increase in lignin content [84]. Similarly, in the leaf elongation zone of darnel ryegrass (*Lolium temulentum* L.), a grass plant, an increase in cell wall peroxidase activity was associated with a decrease in cell expansion during drought stress, due to phenolic cross-linkage formation between cell wall components [85]. However, the association of free phenolic compounds with the cell wall can be an indicator of plant resistance to drought stress [41,81,86]. The introduction of phenolic compounds linked to the cell wall matrix could generate a stiffening of the cell wall, which might alter cell wall elongation after rehydration. The wall is thus more compact, tight, and less permeable to water. This could allow the plant to maintain leaf or root turgor under a low water potential due to the hydrophobic stabilizing properties of these molecules, thus preventing water loss to the apoplast [41,81,86]. In contrast to free phenolics, cross-linked phenolic compounds and lignin could trigger wall rigidification and growth arrest in the later stage of drought stress, which could lead to a loss of productivity, especially for crops [56]. A Diagram summarizing the plant cell wall response to water deficit is presented in Figure 1.

Although much progress has been made over recent years in understanding the consequences of water deficit on cell wall structure and dynamics, much remains to be investigated before targeting key cell wall polymers that could be useful in engineering drought-resistant crops.

## 3. Flooding

The occurrence of floods, which is often associated with climate change, has increased over recent decades. Flooding stress in plants results in a lower oxygen level in the root zone due to this molecule’s low diffusion rate in water [87]. In soybean seedlings and wheat roots exposed to flooding stress, various deleterious effects have been observed, such as suppression or reduction of hypocotyl and root elongation, and suppression of lateral root development. These effects are dependent upon the flooding intensity (water volume and duration) and the extent of recovery periods [88,89,90]. In Arabidopsis root, flooding affected the rapid hyponastic movement of the leaf, which was related to differential growth between the abaxial and adaxial leaf parts [91]. The hyponastic movement could be a locally restricted expansion of the cells in the proximal, abaxial region while adaxial cells remained largely unaffected. *EXP* and *XTH*, whose expression is modulated by SHYG (a NAC transcription factor that regulates 1-aminocyclopropane-1-carboxylic acid oxidase, a key enzyme in ethylene biosynthesis under waterlogging conditions) could contribute to cell wall remodeling, enabling differential growth [91]. Flooding alters cell wall polysaccharides. FTIR analysis performed on maize seedlings exposed to flooding showed a degradation of cell wall polysaccharides and a decrease in pectin content [92]. A similar trend was observed in azuki bean seedling (*Vigna angularis*), where a decrease in cell wall sugar content was observed, correlated with cell wall thinning [93]. Furthermore, the xyloglucan content had a lower molecular mass and the apoplastic pH increased, which might inhibit the activity of expansin [93]. Cell wall polysaccharides play an essential role in maintaining the seedling establishment of an Amazonian plant, *Himatanthus sucuuba*, in floodplains. In the flood zone, the seed endosperm of this tree is richer in mannan while species grown in non-flooded areas contain galactomannan and arabinogalactan, indicating that the presence of galactose on mannan in addition of pectin makes the seed tissues more water-permeable. Thus, the presence of pure mannan slowed down water uptake in seeds adapted to environments with plenty of water [94]. Proteomic analysis performed on soybean seedlings revealed that proteins involved in cell wall metabolism were highly regulated in response to flooding stress [90]. For instance, polygalacturonase inhibitor 2-like protein, expansin-like B1-like and rhamnogalacturonate lyase B-like proteins were flooding-responsive proteins common to all the organs studied (root tip, root without tip and hypocotyl). Polygalacturonase inhibitor 2-like protein and EXPLB1 protein levels were greatly increased, while the abundance of other cell wall-related proteins, including cellulose synthase-interactive protein 1 and CAD, decreased, which was dependent on the severity of the flooding. In wheat roots under flooding stress, similar effects to those observed with soybean have been reported. Most down-regulated proteins, such as β-glucanase, β-glucosidase, and β-galactosidase, are related to cell wall metabolism and structural modification [88]. It has been suggested that these down-regulated proteins might be correlated with the inhibition of cell wall elongation under flooding stress, which significantly suppressed the growth of wheat plants.

These alterations in protein abundance are regulated by transcriptional changes since RT-qPCR analysis showed that flooding treatment resulted in the down-regulation of genes encoding cellulose synthase in soybean roots; this could have consequences on cellulose synthesis [95]. Similar trends were observed in woody plants such as gray poplar where the expression of genes encoding cellulose synthases, required for secondary cell wall formation, was strongly down-regulated (up to 44-fold) in roots, while those involved in primary cell wall biosynthesis were less drastically affected (up 4.4-fold). These effects were dependent upon the period of flooding exposure. The primary wall would therefore be a key target for mediating cellular integrity in response to flooding [96]. In addition, other genes encoding proteins involved in cell wall degradation, including cellulase, β-(1,4) glucanase, pectate lyase and polygalacturonase, were down-regulated suggesting the reduced enzyme activity of these proteins could alter the cell wall structure with consequent effects on root growth, leading to growth arrest and suppression of lateral root formation [95]. Another transcriptome analysis performed on soybean seedlings, showed differential mRNA abundance of PGIP and expansin encoding genes. The discrepancy between the inhibition of cell elongation of soybean seedling under flooding and the relative increase in the abundance of EXPLB1 could indicate that flooding recruits proteins with distinct functions in the cell wall. To a certain extent, PGIP, expansin and rhamnoglacturonate lyase B could be flooding stress indicator proteins, which could trigger defense systems [90].

Flooding alters lignification as shown by the decrease in lignin deposition, assessed by phloroglucinol staining, being suppressed in roots and hypocotyls of soybean seedlings under flooding stress [97]. In parallel, proteomic analysis showed a down-regulation of an extracellular superoxide dismutase (Cu-Zn), which might play a role in the regulation of lignification under flooding. Germin-like protein with oxalate oxidase or superoxide dismutase activity produces H_2_O_2_, which could be used in cross-linking reactions within the cell wall. Four germin-like protein precursors were down-regulated during flooding, thus indicating a reduction in the linking of polysaccharides and proteins within the cell wall, as well as in lignification [97]. In addition, in poplar, the expression of genes encoding cell wall degradation enzymes, such as genes encoding lignin biosynthesis enzymes, including PAL, trans-cinnamate 4-hydroxylase (C4H), 4 coumarate CoA-ligase (4CL), ferulate 5-hydroxylase (F5H), COMT, and CCoAOMT, were repressed indicating the alteration of lignification biosynthesis under flooding.

**Figure 2:**
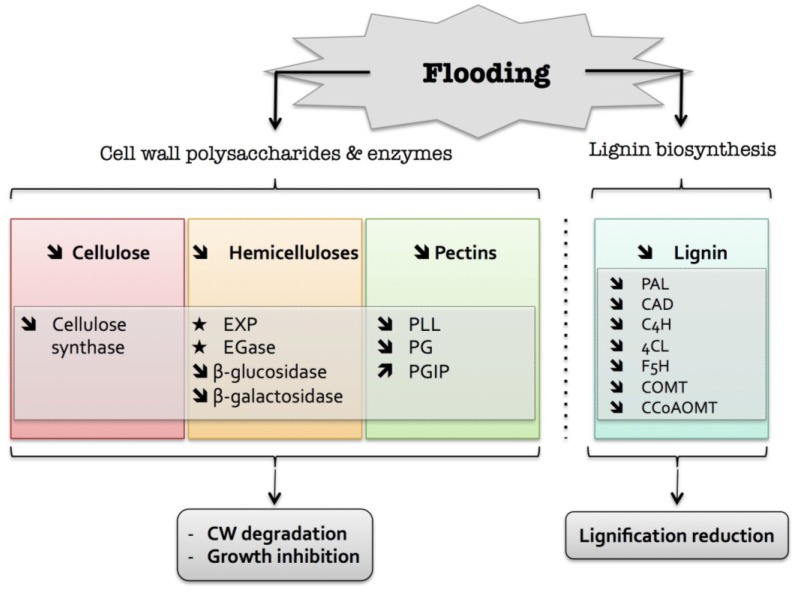
Overview of the plant cell wall response to flooding. The schematic presentation is deduced from the results of different studies reported in the review. Arrow (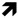) means increased abundance and arrow (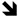) means decreased abundance of the molecules. Star (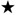) means contrasting data on the gene/protein or the molecule studied according to the literature cited in the review. Cell wall (CW); expansin (EXP); endoglucanase (EGase); polygalacturonase (PG); polygalacturonase inhibitor protein (PGIP); pectin/pectate lyase-like (PLL); phenylalanine ammonia-lyase (PAL); hydroxycinnamyl alcohol dehydrogenase (CAD); trans-cinnamate 4-hydroxylase (C4H); 4 coumarate CoA-ligase (4CL); ferulate 5-hydroxylase (F5H); caffeate *O*-methyltransferase (COMT); caffeoyl-CoA 3-*O*-methyl-transferase (CCoAOMT).

These data are mostly derived from genomic or proteomic approaches and provide valuable knowledge about the role of the plant cell wall in response to oxygen deprivation (Figure 2). However, very few data are currently available at the biochemical level on cell wall polymers, cell wall enzyme activities and signaling processes that regulate this response.

## 4. Heat

In the context of global warming, an increase in temperature is likely to be a key abiotic stress to which plants will have to adjust to survive [98]. Heat stress is often considered an increase in temperature beyond a threshold level for a period of time that induces irreversible damage to plant growth (*i.e.*, 10–15 °C above the basal temperature). The upper threshold temperature depends on the plant species and genotypes within the species (for a review: [98]). Heat stress is an important agricultural problem in many areas, which can lead to a drastic reduction in economic yield, especially for cereals [99,100]. The application of transitory or constantly high temperatures causes a range of morpho-anatomical, physiological and biochemical changes in plants, particularly observed in Arabidopsis, maize and wheat [101,102,103,104]. These affect plant growth and development but in a different way, depending on the developmental stage at which plants are exposed [99]. Various studies have demonstrated that roots are more sensitive to heat stress, suggesting that high soil temperature is more detrimental than high air temperature for whole-plant growth [105]. To maintain growth and productivity, specific tolerance mechanisms must be implemented by plants [106]. Although the cell wall is not the structure primarily altered under heat shock, some studies have revealed that it could be affected at various levels. Cell wall composition and sugar metabolism were compared between maize genotypes during corn expansion, as a target for genetic improvement during heat stress [107]. In sweet corn plants, sugar concentrations in both hemicellulose and cellulose fractions of cobs decreased under stress treatment. The hemicellulose fraction in the shank from the first ear also decreased. In contrast, in dent corn plants, there was no reduction in sugar content, except in the cellulose fraction. Overall, this suggested that synthesis of cell wall components was affected by heat stress and differed between genotypes. High temperature treatment enhanced growth of vegetative plant parts but reduced husk expansion, by suppressing cob extensibility through impairing hemicellulose and cellulose synthesis following a reduction in photosynthesis supply. Therefore, the reduction in grain yield after high temperature treatment was due to effects on sink rather than on source activities [107]. In coffee leaves, heat treatment (37 °C) generated changes in cell wall polymers, as illustrated by a ~40% increased level in leaf hemicelluloses and a 50% decreased level in pectin, with contrasting effects depending on the sugars considered [108]. For instance, in the pectic fraction, arabinose and galactose contents were increased by 40% and 44% respectively, whereas uronic acid and rhamnose decreased by 60% and 40% respectively. An increase in arabinose and galactose contents could be related to a larger amount of type II arabinogalactan, which could contribute to the rigidification of the cell wall by either oxidative cross-linking or water retention. In contrast, the decrease in rhamnose and uronic contents probably reflected changes in the structural organization of cell wall pectin. In the non-cellulosic fraction, the increased xylose content (~30%) was associated with a decrease in arabinose (~33%), thus indicating a lower substitution of arabinose in the xylose backbone of xylan under heat stress. Furthermore, the analysis of leaf apoplastic proteomes of *Coffea arabica* plants reflected these changes in cell wall metabolism in a changing environment related to the greenhouse effect [109]. In contrast, in wheat grain, which displays a primary type II cell wall, arabinoxylan content increased under heat stress [110]. Overall, this shows that the plant response might be dependent upon its cell wall type. Cell-wall related gene expression can be altered in response to heat stress as well. A transcriptomic study performed on Chinese cabbage (*Brassica rapa* L.) showed that some cell wall-related genes could play a role in the acquisition of thermotolerance [111]. Indeed, several genes, encoding proteins from the XTH family, were up-regulated following heat treatment. Other genes, encoding cell wall enzymes or proteins as diverse as arabinogalactan protein, β-glucosidase, cellulose synthase, expansin, extensin, glycosyl transferase, pectin esterase and xylosidase, were up-regulated up to 2–3 fold following their first exposure to permissive high temperature conditions (37 °C). This corresponds to acclimation to a non-lethal temperature to acquire thermotolerance, *i.e.*, the ability for the plant to survive an otherwise lethal high temperature. These results support the idea that thermotolerance acquisition partially requires cell wall-remodeling enzymes, which could be involved in the biosynthesis or *in muro* modification of major cell wall polymers [111]. The authors suggested that the functional significance of XTH during this period could correspond to establishing secondary cell wall deposition, after cell elongation has ceased. Following high temperature exposure, these proteins may have a role in wall strengthening, in addition to that classically observed for cell expansion, which might help plants to adapt to high temperature. XTH could be involved in the reinforcement of connections between the primary and secondary cell walls, thus increasing XG polymerization. In another study, performed on grapevine during fruit ripening, an increased level of *XET* transcripts was measured following heat stress [112]. This could be related to the adaptation of berry volume to temperature and the need for more flexible cell walls. As observed for water stress, the fine-tuning of the cellulose-hemicellulose network appears to be a key component of heat tolerance. In *Agrostis* grass species, expansin is one of the major genes that is up-regulated in response to heat stress at 40 °C [113]. Cell walls with increased levels of expansin may therefore be loosened, relaxed, and become more elastic, which could help maintain cellular functions during heat stress. The overexpression of a grass expansin gene, *PpEXP1* from *Poa pratensis*, in tobacco plants led to a better heat tolerance (35 °C), with less structural damage to cells. This confirms that expansin plays an important role in plant adaptation to heat stress [114]. However, it appears specific to species and/or expansin isoforms, as in *Brassica napus* seedlings subjected to heat stress, where the expression of *EXPA5* was down-regulated up to 10-fold compared to the control [115]. Similar results were observed in poplar (*Populus simonii)*, where thirteen genes encoding *EXP* were down-regulated [116]. Other cell wall-remodeling enzymes are regulated under heat stress at gene expression level, such as a PME gene, *PME35*, which was reduced nearly 10-fold in *Brassica napus* [115]. 

In addition to its effects on the primary cell wall, heat stress can mediate changes in secondary cell wall metabolism. Synthesis of lignin can be altered during the application of elevated temperature. For instance, in *Coffea* leaves, heat stress increased G and S monolignol contents while a decrease in H monolignol was observed, indicating a structural change in lignin [108]. A proteomic analysis on rice seedlings, growing under various high temperatures, highlighted a differential expression of proteins involved in lignin biosynthesis, including PRX and PAL, with consequent effects on the inhibition of lignification [117]. In contrast to these results, a gene encoding a laccase was up-regulated in Chinese cabbage under heat treatment [113]. This enzyme is suspected of being involved in lignin biosynthesis, based on its ability to oxidize lignin precursors. This result is in accordance with observations in strawberry where elevated temperature possibly regulated the lignin biosynthetic pathway through increased PRX activity [118].

**Figure 3:**
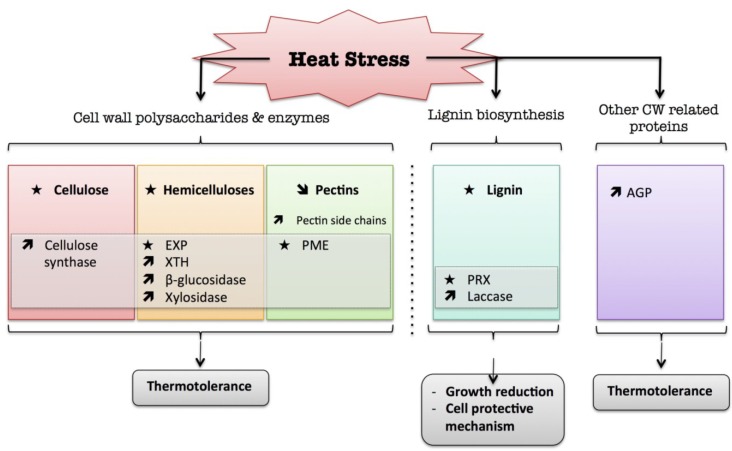
Diagram summarizing the plant cell wall response to heat. The schematic presentation is deduced from the results of different studies reported in the review. Arrow (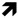) means increased abundance and arrow (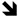) means decreased abundance of the molecules. Star (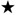) means contrasting data on the gene/protein or the molecule studied according to the literature cited in the review. Cell wall (CW); xyloglucan endo-β-transglucosylases/hydrolases (XET/XTH); expansin (EXP); pectin methylesterase (PME); cell wall peroxidases (PRX); arabinogalactan protein (AGP).

Heat causes changes in cell wall metabolism and this is an important physiological mechanism of heat tolerance (Figure 3). However, much more work is needed to gain a clearer understanding of the cell wall at the physiological, genetic, and biochemical levels of plant heat tolerance.

## 5. Cold

Cold stress, which includes both chilling and freezing injuries, modifies gene expression and plant metabolism with consequent effects on many biological functions [119,120,121,122]. The aerial parts of the plant are affected, including leaf expansion, wilting, chlorosis, and necrosis [123,124]. Root growth can also be altered under cold stress [125]. During drastic cold exposure, freezing can induce extracellular ice formation, which leads to cell dehydration and may result in cell collapse [126]. This could be related to the mechanical constraint generated by the enlargement of ice crystals on the cell wall and the plasma membrane, ultimately leading to cell rupture [123,127]. In relation to global warming, the seasons are often altered with changes in temperature and occurrence of freezing stress that can appear without any previous chilling period [128]. A sudden shift to extreme temperature can induce severe physiological damage, which is more important for native plants of a warm habitat cultivated in a temperate climate, most of which are of agronomic interest (maize, soybean, tomato, *etc.*). Such stress can cause a marked decrease in production and significant economic loss [124]. The challenge is thus to improve crops to winter hardiness [129]. In the past twenty years, major efforts have been directed towards understanding the plant response to chilling (0–15 °C) and frost (<0 °C) temperatures [121,127,130,131,132,133]. Temperate plants are often chilling-tolerant. They have also developed mechanisms to increase their ability to withstand freezing temperatures following a period of low but non-freezing temperatures [134,135,136,137]. This process, called cold acclimation, is a multigenic and quantitative trait that is associated with complex physiological and biochemical changes [119,137] (for a review: [138]). Aside from other factors such as membrane integrity and protoplast properties, plant resistance to cold stress often depends on tissue morphology and cell wall mechanical properties [139,140,141,142]. It has been proposed that cell wall rigidity may be an important factor in cell resistance to freeze-induced dehydration [143].

Cold acclimation induces changes in cell wall polysaccharide composition and in the activities of cell wall-modifying enzymes. In a suspension culture of grape cells or in apple, cold acclimation increased cell wall strength and decreased the pore-size of the cell wall [143]. Pectins appear to be a key element of the plant response to cold stress, as shown by a number of studies on various species. In oilseed rape leaves, the cold response was associated with an increase in pectin content [142,144]. This was also observed in pea epicotyls during cold acclimation (2 °C), with a higher level of arabinosyl residues, and in bromeliad (*Nidularium minutum*), a tropical plant, where pectin content increased in plants grown at 10 °C and 15 °C compared to those grown at 25 °C [145,146]. In a frost-tolerant pea genotype, cold acclimation (10 °C day/2 °C night) was accompanied by an increase in homogalacturonan (HG), xylogalacturonan and highly branched rhamnogalacturonan I with branched and unbranched (1→5)-α-arabinans and (1→4)-β-galactans. HG was more esterified in the frost-tolerant pea genotype. The increased cold tolerance in the frost genotype might be related to an increased synthesis of arabinan and galactan side chains and HG methylesterification status, which may act as a gelling component, or regulators of pore size as well as a cold protectant following frost exposure [147]. Changes in the activities of pectin-modifying enzymes have been shown for several species in variable growth conditions in response to cold. For instance, increased PME activity was observed in cold-acclimated oilseed rape plants, which correlated with an increased rigidity of the cell wall in the leaf. This rigidity could be related to a demethylesterification of pectin, which generates free carboxyl groups, and Ca^2+^ ion-mediated cross-linking of these carboxyl groups to create a stiff pectate gel [142]. In contrast, in two chicory root varieties subjected to low temperature in field experiment, PME activity was differentially affected [148]. PME activity was higher during summer and then decreased consistently in both varieties (Nausica and Arancha). However, the decrease in PME activity was stronger in the Nausica variety, which is more cold-sensitive, compared to the Arancha variety where PME activity almost recovered by the end of the cold season [148]. Unlike in oilseed rape, PME activity was not increased in a frost-tolerant pea genotype [147]. During pollen germination, the expression levels of many genes responsible for pollen germination and tube growth were not influenced by cold stress, in particular PME, which were strongly expressed under normal conditions and had steady-state levels of expression in cold-treated samples [149]. This discrepancy could indicate distinct genotype-, species- or tissue-dependent mechanisms of temperature control of PME activity. PG is also regulated in response to cold stress. In young rice leaves of *Ubi::OsBURP16* transgenic plants, which overexpressed a beta-subunit of PG, reduced cell adhesion caused by pectin degradation, which affected cell wall integrity, was associated with a decreased tolerance to cold stress [76]. Cold treatment does not only affect pectin-remodeling enzymes but also pectin-associated receptors or WAKs (wall-associated kinases). WAKs are required for cell elongation and development and were shown to play a role in the plant/pathogen interaction [38,150,151,152,153]. An up-regulation of *WAK* genes was observed in rice seedling and a cold-tolerant maize genotype under cold treatment [154]. WAKs could bind either oligogalacturonides or pectin in the cell wall in response to cold stress, indicating a potential role of the cell wall as a cold stress sensor [154].

This modification in pectin structure is often accompanied by changes in neutral sugar composition in vegetative tissues [126,145]. An increase in hemicellulose content could enhance cell wall stiffening and prevent cell collapse caused by dehydration [144,155]. A reduction in xylose and glucose contents in non-cellulosic components was observed in cold-acclimated winter oilseed rape leaves [144]. In contrast, in the winter/dormant stems of poplar, xylose and glucose contents increased and the cellulose content increased by almost 3-fold [156]. Unlike a frost-tolerant pea genotype, a frost-sensitive pea cultivar accumulated substantial amounts of (1→4)-β-xylans and glucuronoxylans, but not pectins, indicating a premature end to cell growth and an initiation of vascularization [147]. Some alterations in the type II cell wall have been shown upon cold exposure. In Miscanthus plants grown at the juvenile stage, an increase in (1→3),(1→4)-β-D-glucan content was observed during cold acclimation (12 °C day/12 °C night) while in wheat roots cultivated over a low temperature acclimation (2 °C) period, hemicellulose synthesis was modified [155,157]. Hemicellulose synthesis increased during the first 24 h following application of stress. In contrast, in the first hour of low temperature exposure, a decrease in the proportion of β-glucans and branched glucuronoarabinoxylans occurred while an increase in the activities of β-fucosidase and β-glucosidase was observed. The activities of glycosidases could account for the transient accumulation of GXAG (oligosaccharin) in winter wheat tissues during the first hours of cold acclimation at 2 °C [158]. Such an oligosaccharin could be produced as a result of the increased hemicellulose turnover and could be physiologically active oligosaccharides that could stimulate the acquisition of freezing tolerance in a winter variety of *Triticum aestivum* L. [155]. Moreover, the authors hypothesized that GXAG could increase cell receptivity to ABA signaling [155,158].

The observed changes in cell wall composition correlate with a strong up-regulation of genes encoding proteins involved in cell wall modification [155]. During cold acclimation, *EXP* and *XTH* gene expression was down-regulated in sweet potato while *EXP* genes were up-regulated in Arabidopsis and rice, showing a contrasting regulation of these genes at low temperatures [125,159,160]. In a distinct experiment, extensin and *XTH* encoding genes were down-regulated in Arabidopsis under cold response [161]. The functional role of XTH was shown in Arabidopsis transgenic plants, overexpressing *AtXTH21*, which had an improved freezing tolerance [162]. Similarly, in rice, a xyloglucan endotransglucosylase (*OsXET9*) was highly induced under cold stress, especially in seedlings [163]. Transcriptomic data in cotton showed an up-regulation of genes encoding a cellulose synthase, *PME3*, and *MUR4*, a UDP-D-xylose 4-epimerase, an enzyme that catalyzes UDP-xylose-4-epimerase to UDP-L-arabinose, in response to cold [164]. In potato plants, the expression of xylose isomerase, which catalyzes the conversion of D-xylulose into D-xylose, a constituent of non-cellulosic polysaccharides, was increased [165]. Overall, cold stress had a drastic effect on the regulation of genes encoding cellulose synthase, as illustrated by the results obtained on cotton, in which cellulose synthases were down-regulated by 2-fold upon cold treatment [164]. In poplar during the winter dormancy period, cellulose synthesis was increased in stems compared to summer stems [156,166], but most cellulose synthase genes were surprisingly down-regulated, which might suggest an unknown control mechanism of cellulose deposition in the winter cell wall of stems [156]. In contrast, in a rice cold-tolerant genotype, cellulose synthase genes were up-regulated by 5 to 8-fold after 48h of cold exposure [154]. The effects of cold stress can also target other cell wall compounds such as arabinogalactan proteins (AGPs), which are located in plant cell walls and belong to a super family of highly glycosylated hydroxyproline-rich glycoproteins. In cotton, the expression of a non-classic AGP which contains a predicted signal peptide, a short AGP domain of seven amino acids, a His-stretch, a Pro-rich domain and a PAC (PRP-AGP containing Cys) domain, was found to be up-regulated in response to cold stress [167]. Transgenic Arabidopsis plants overexpressing *GhAGP31* confirmed that GhAGP31 improved the freezing tolerance of Arabidopsis seedlings [167].

Lignification and changes in lignin content can occur during growth at low temperature as well [23]. Lignin synthesis can be enhanced during cold acclimation and participate in cell wall modification by strengthening it, thus preventing freezing damage and cell collapse. A large increase in CAD (hydroxycinnamyl alcohol dehydrogenase) activity under cold stress was observed in frost-sensitive or cold-tolerant Miscanthus genotypes, but it was largest in the frost-tolerant clone *M. sinensis* August Feder [157]. Furthermore, PAL enzyme activity was higher in the frost-tolerant genotype during cold acclimation [157]. In *Brassica napus*, PAL activity, as well as the rate of accumulation of different phenolic compounds in leaves, depended on the range of low temperature applied to the plant [168,169,170]. The accumulation of ferulic acid observed in these leaves could increase cell wall rigidity and cell resistance to mechanical stress, thus explaining the growth delay by limited cell expansion [23,170,171]. These data suggest that the phenolic pathway activated by cold stress participates in protecting against freezing damage [23,157]. Lignin biosynthesis genes are affected in the cold response. It was shown that *CAD* gene expression was induced in sweet potato or Arabidopsis in response to cold [161,172]. Other genes involved in lignin biosynthesis, including *PAL*, *CCR*, *COMT* (caffeate *O*-methyltransferase), and *CCoAOMT*, were shown to be up-regulated in pea and *Phaseolus vulgaris* in response to cold [161,164,173,174]. This is concomitant with the increase in cell wall strengthening in response to low temperature stress.

**Figure 4:**
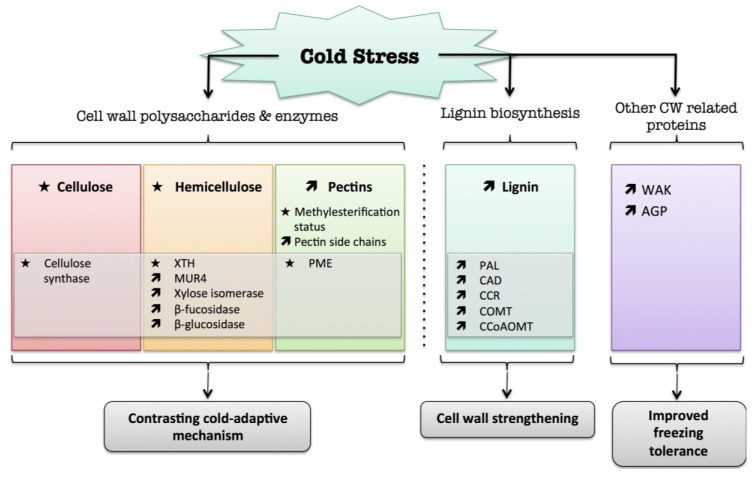
Overview of the plant cell wall response to cold. The schematic presentation is deduced from the results of different studies reported in the review. Arrow (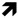) means increased abundance and arrow (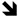) means decreased abundance of the molecules. Star (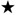) means contrasting data on the gene/protein or the molecule studied according to the literature cited in the review. Cell wall (CW); xyloglucan endo-β-transglucosylases/hydrolases (XET/XTH); pectin methylesterase (PME); UDP-D-xylose 4-epimerase (MUR4); phenylalanine ammonia-lyase (PAL); hydroxycinnamyl alcohol dehydrogenase (CAD); caffeate *O*-methyltransferase (COMT); caffeoyl-CoA 3-*O*-methyl-transferase (CCoAOMT); cinnamoyl-CoA reductase (CCR); cell wall peroxidases (PRX); arabinogalactan protein (AGP); wall-associated kinase (WAK).

All these data show that the cell wall structure changes in response to cold (Figure 4). Although it is likely that the cell wall could modulate different aspects of the cold response, an investigation of the cell wall as a primary effector is still required.

## 6. Salt 

Another major abiotic stress is due to soil salinity, which can affect plant productivity. More than 6% of the world’s total land and approximately 20% of irrigated land is affected by this abiotic stress [175]. Increasing salinization of arable land is expected to have drastic effects, with up to 30% land loss within the next 15 years [106]. Salt stress has two origins: the deposition of oceanic salts by wind and rain and the erosion of rocks that release soluble salts [175,176]. The major effect of salt stress is a decrease in available soil water due to the reduction in osmotic potential of the soil solution leading to water deficit-related plant growth impairment [177,178,179]. Plants display shoot growth inhibition during the first phase of salt stress, partly due to a loss of cell wall extensibility. In maize, reduced cell wall extensibility was related to the formation and accumulation of diferulic acid cross-links in the cell [180]. Changes in cell wall structure appear to be a common feature of the response to salt stress. For instance, in coffee plants grown with 150 mM NaCl during 25 days, the palisade and spongy parenchyma exhibited thinner cells, which might be related to decreasing water content in leaf cells [181]. Such physiological response was reported in salt-sensitive plants, called glycophytes. This group includes a number of cultivated plants such as rice, soybean and maize [180,182,183]. The resistance of glycophytes to lethal salinity can be improved by short exposure to low levels of salt stress, a process known as salt acclimation [177,184]. This acclimation mechanism has been described and shown to involve changes in cell wall-related proteins [185,186]. Candidates that could participate in salt acclimation are receptor-like protein kinases (RLKs), primary cell wall “sensors”, such as WAKs [187]. Although in abiotic stress the function of this protein is not so well defined, induction of *WAKL4* (WAK-like kinase 4) gene expression was observed in Arabidopsis roots under high salt concentration (750 mM NaCl) [188]. These data suggest that the main salt stress perception-to-signaling event could occur at the cell wall-plasma membrane interface. Following salt stress perception, the plant response was shown to involve the regulation of a number of primary cell wall protein-encoding genes, through the recruitment of specific transcription factors. For example, *AtMYB41* or the rice R2R3-type MYB transcription factor *OsMPS* (MULTIPASS) were activated by salt stress (100 mM NaCl) and shown to regulate the biosynthesis of cell wall-related genes [189,190]. *OsMPS* is transiently expressed in vegetative and reproductive tissues and negatively regulates the expression of expansin and endoglucanase genes under salt stress. The hormone-dependent regulation of *OsMPS* expression allowed root growth adaptation to changing environmental conditions while *AtMYB41* was shown to down-regulate the expression of *EXP* or *XTH* encoding genes and up-regulate the expression of *HRGP* when Arabidopsis plants were exposed to 200 mM NaCl [191]. There appear to be distinct responses when species and/or salt-stress conditions are compared. For instance, *RhEXPA4* expression in rose was increased by salt while transgenic Arabidopsis plants overexpressing *RhEXPA4* displayed a compact phenotype (shorter stems, curly leaves, compact inflorescence) and were salt-tolerant [67]. Similar results were observed with the overexpression of wheat expansin, *TaEXPB23*, in transgenic tobacco plants [192]. However, Arabidopsis transgenic plants overexpressing *AtEXP3* or *AtEXP-B1* were salt-sensitive following exposure from 250 mM to 300 mM NaCl treatment [193]. In physic nut roots (*Jatropha curcas*), *EXP* genes were both up-regulated and down-regulated while *XET* genes were up-regulated during different periods of salt exposure at 100 mM NaCl [194]. In parallel, other structural cell wall proteins, including AGP and proline-rich protein (PRP), were affected by salt [25,195]. The expression of rice and poplar (*Populus euphratica*) genes encoding AGP and PRP was up-regulated in response to salt [196,197,198,199]. The glycine-rich proteins (GRP), another class of cell wall structural proteins, appear to be involved in salt tolerance. Genes encoding these proteins were induced under salt exposure, with consequent effects on seedling growth and seed germination [200]. MsGRP, a GRP from *Medicago sativa*, probably participates in iron or water transport to maintain cellular homeostasis [200]. *SOS6*, which encodes a cellulose synthase-like protein *AtCSLD5*, plays a role in the regulation by decreasing ROS content under salt stress. The Arabidopsis *sos6* mutant displayed a decreased level of arabinose, rhamnose and galacturonic acid sugar contents under salt stress suggesting that SOS6 is required to maintain cell wall integrity in these conditions [201]. In the primary cell wall of glycophytes, other classes of proteins and polysaccharides are modified upon salt stress treatment. These include RGP (reversibly glycosylated polypeptide), such as RGP1 in pea roots (*Pisum sativum*), or glycosyl hydrolase 1 (GH1 family), GRP in Arabidopsis root, which are up-regulated under salt treatment [200,202,203,204]. In potato, an increased expression of a UDP-glucose-4-epimerase (UGE), which interconverts UDP-glucose and UDP-galactose, was observed under various abiotic stresses such as high salinity [165]. Moreover, in red-osier dogwood seedlings, a salt-adapted plant, the hemicellulose composition and the lignification status were not altered but an increase in the rhamnose content in the pectic fraction was observed, accompanied by a decrease in plant growth [205]. In addition, cellulose and hemicellulose displayed a steady-state level while a slight decrease in uronic acid was observed in root of a salt-tolerant soybean genotype in response to salt stress. In the root elongation zone of the salt-sensitive soybean genotype, a significant decrease in the cell wall total sugar content was shown, with a more severe decrease (~70%–80%) in the abundance of pectin, compared to that observed in the salt-tolerant one which only displayed a 30% decrease [206]. The higher abundance of pectin in the salt-tolerant cultivar could explain its salt tolerance. A similar study performed on three maize hybrids with contrasting salt tolerance showed a decrease in cellulose concentration in all genotypes and an accumulation of non-methylated uronic acid, which was more pronounced in the salt-sensitive maize genotype [207]. This accumulation was delayed in the salt-tolerant maize genotype, particularly in the youngest shoots, which favored their elongation due to the smaller increase in non-methylated uronic acid [207]. Unlike maize, highly methylated pectin, as shown by immunolabeling with JIM7, increased within the cell wall of Aspen petiole cells [208]. This was associated with an increase in the elasticity modulus, indicating a decrease in cell wall plasticity to maintain the turgor pressure necessary for plant growth. According to these authors, the increase in highly methylated pectin could prevent water molecules from binding to pectin leading to a less hydrated cell wall, and thus favoring water dehydration in the plant during salt stress.

Salt stress has been shown to impact secondary cell wall formation and structure, as revealed by an altered lignin biosynthesis. Salt treatment increased root lignification and the number of lignified vessels [182,209]. Many enzymes involved in the lignin biosynthesis pathway were altered upon salt treatment. For instance, in tomato, *SlPAL5* gene expression was increased after treatment with 200 mM of NaCl while the expression of PAL was decreased in rice [210,211]. Proteomic studies have also revealed that COMT and CCoAOMT are salt-responsive proteins [26]. This was confirmed at the activity level as in rice roots, COMT enzyme activity was decreased in salt stress conditions [211]. However, the CCoAOMT enzyme was salt-induced in the roots of Arabidopsis and rice [212,213]. The action of these enzymes might help reduce the bypass water flow that allows Na^+^ ions to enter rice roots *via* an apoplastic pathway [214].

The response of halophyte plants to salt stress appears to differ from that of glycophytes. Indeed, in these plants, cell homeostasis and osmotic adjustment are maintained, with consequences on the synthesis of proteins and cell wall pectins [215]. For instance, in *Sonneratia alba* (a halophyte plant), a higher calcium concentration was measured suggesting that the plant could sequester and efficiently use Ca^2+^ within cell walls to increase their rigidity through pectin egg-box formation [216]. Like in glycophytes, WAK protein could be a salt-stress sensor. In favor of this hypothesis, the results obtained in *Nitraria sphaerocarpa* showed that the expression of *WAK4* was increased after long exposure to salt [217]. Salt also has consequences on proteins that are involved in the biosynthesis or remodeling of cell wall polymers. In roots of *Salicornia europaea*, genes encoding cell wall proteins of the primary cell wall, including UDP-L-rhamnose synthase and cellulose synthases, were down-regulated while other genes, encoding pectin methylesterase inhibitor proteins, were increased under saline conditions [218].

**Figure 5:**
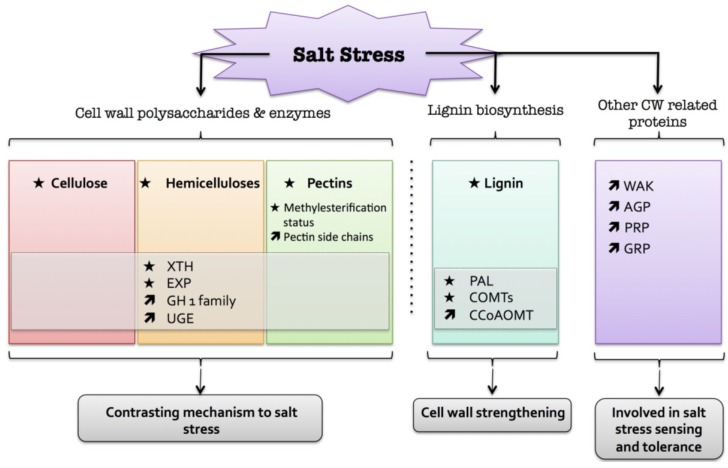
Diagram summarizing the plant cell wall response to salt. The schematic presentation is deduced from the results of different studies reported in the review. Arrow (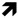) means increased abundance and arrow (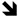) means decreased abundance of the molecules. Star (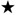) means contrasting data on the gene/protein or the molecule studied according to the literature cited in the review. Cell wall (CW); xyloglucan endo-β-transglucosylases/hydrolases (XET/XTH); glycosyl hydrolase family (GH); reversibly glycosylated polypeptide (RGP); phenylalanine ammonia-lyase (PAL); caffeate *O*-methyltransferase (COMT); caffeoyl-CoA 3-*O*-methyl-transferase (CCoAOMT); arabinogalactan protein (AGP); wall-associated kinase (WAK); proline-rich protein (PRP); glycine-rich protein (GRP).

In conclusion, the cell wall plays an important role in salt tolerance, especially in the detection of salt stress. However, the cell wall modifications are different in glycophytes and halophytes. In glycophytes, there is an increase in primary and secondary cell walls whereas in halophytes only the secondary cell wall structure is altered (Figure 5).

## 7. Heavy Metals

Heavy metal (HM) pollution has become an increasingly serious environmental problem over recent decades due to its toxicity, leading to many detrimental effects on living organisms, from plants to animals [219,220]. For instance, heavy metal exposure was shown to reduce root growth and to enhance formation of lateral roots [221,222,223]. When considering plant mineral nutrition, metals are often categorized into those that are essential for many physiological processes, e.g., copper (Cu), zinc (Zn) and iron (Fe), and non-essential ones, such as lead (Pb), cadmium (Cd), aluminum (Al) and mercury (Hg). An excess, as well as a deficiency, of the former can cause harmful effects in plant cells, while the latter may be toxic even at relatively low concentrations [224]. It is also possible to classify them as redox-active metals (e.g., Cu and Fe), which catalyze the formation of hydroxyl radicals by directly participating in the Haber-Weiss reaction, or metals without a redox capacity (e.g., Cd, Pb, Hg, Zn) which inactivate the cellular antioxidant pool and disrupt the metabolic balance, thus enhancing the load of ROS [225,226]. This affects the distribution of calcium (Ca^2+^), Ca^2+^-binding protein activity, signaling processes and MAPK pathways [227,228].

The cell wall represents a physical barrier against the entry of HM into the symplastic compartment. One strategy of plant cells for coping with HM is to remove them from the protoplast by sequestration into extra-cytoplasmic compartments such as the cell wall [27,28]. This protects the most sensitive sites within the protoplast from HM toxicity [27,229,230]. Plant cell wall polysaccharides play a crucial role in HM binding and accumulation in the cell wall, although other compounds, such as proteins, amino acids and phenolics, can also take part in this process [27,231]. One of the advantages of precipitating metals within the cell wall is their strong metabolic inactivation [232].

Pectin, together with hemicellulose, plays a crucial role in binding HM [233,234]. It was shown that the presence of pectin containing boron cross-linked rhamnogalacturonan II in the cell wall promoted the formation of an Al-complex, preventing damage and the irreversible inhibition of growth [235,236]. The presence of exogenous silicon increased cell wall affinity for Zn in maize roots and rice seedlings, thereby reducing its uptake and translocation [237,238]. The degree of methylesterification of pectins appears to contribute to HM tolerance [239]. Some amounts of heavy metal bound to low-methylesterified pectins in the cell wall can enter the protoplast together with this compound by endocytosis, in particular in meristematic dividing cells [229,240]. A striking example of metal accumulation in the cell wall was observed in the common reed (*Phragmites australis)*, which is considered to be a plant with a high detoxification and phytoremediation potential. Zn concentrations followed a gradient with the sequence: intercellular space >cell wall > vacuole > cytoplasm, indicating that most Zn was accumulated within the apoplast [232]. The ability to bind divalent metal cations depends on the number of functional groups, such as –COOH, –OH, and –SH present in the cell wall [241]. The main pectic domain responsible for binding divalent and trivalent metal cations is HG. The HG fraction, particularly those with a low degree of methylesterification, recognized by JIM 5 antibodies containing some amounts of free carboxyl groups, can bind divalent and trivalent metal ions [242]. It is known that low methylesterified HG can interact with calcium and form an egg-box structure in the cell wall. Calcium ions can be replaced by trivalent Al^3+^, and also by divalent HM cations, including Pb^2+^, Cu^2+^, Cd^2+^ and Zn^2+^ [243]. For instance, under short-term Al supply, Al accumulated primarily in the root apoplast where Al^3+^ strongly binds to the negatively-charged binding sites provided by non-methylesterified pectin in the cell wall [239]. In addition, in flax hypocotyls, Cd^2+^ caused reorganization in the distribution of pectin epitopes related to PME activities [244]. Enhanced JIM 5 and 2F4 labeling at the junctions of the inner tissues indicated that the presence of blockwise de-esterified HGA might oppose cell separation under Cd stress [245,246]. This was accompanied by an increase in PME and peroxidase enzyme activities, indicating a reinforcement of cell junctions leading to better tissue cohesion and Cd sequestration [246]. An increase in cell wall polysaccharides confers on the plant the capacity to store metal in the cell wall. In the common bean root apex, the reduction in pectin in the cell wall following PEG treatment is likely to change cell wall structure, thus affecting its porosity and inhibiting Al accumulation [247]. In another study, Al induced pectin biosynthesis in the roots of an Al-tolerant rye cultivar, but reduced it in the sensitive one [248]. This was associated with a decreased level in root PME gene expression in the tolerant rye genotype, although Al treatment increased PME activity in both cultivars.

The degree of methylesterification and acetylation of pectin decreased its affinity for HM. In a copper-tolerant plant, *Silene paradoxa*, a reduction in the root pectin content, together with an increase in its methylesterification status, was observed, in contrast to the copper-sensitive cultivar, indicating a Cu-tolerant/excluder phenotype [249]. In *Medicago truncatula*, the silencing of two Al-induced genes, pectin acetylesterase and annexin, in hairy roots slightly increased the sensitivity of the plant to Al, suggesting that these genes play a role in Al resistance [250].

In addition to pectin-remodeling enzymes, other cell wall enzymes are regulated following HM exposure. For instance, in Arabidopsis, XET enzyme activity was inhibited under exposure to Al, which was mostly bound to hemicellulose [251]. This was associated with root growth inhibition. Interestingly, transgenic poplar overexpressing *XET* had a higher Cd tolerance by reducing Cd uptake and accumulation in roots. This was accompanied by a higher xyloglucan degradation activity, leading to a reduction in XG content in the root cell wall [252].

The structure of the cell wall has an effect on the translocation of HM within xylem. In rice plant, mutation in a cellulose synthase gene (*CESA9*) modified the structure of the secondary cell wall, which further reduced translocation of Cd within xylem and its accumulation inside the plant [253]. In *Elsholtzia splendens*, a hyperaccumulator of Cu, excess Cu altered the composition and distribution of cell wall sugars in roots [254]. Cu enhanced cellulose, hemicellulose and pectin contents with a particular increase in galacturonic acids and KDO levels during HM exposure. In *Sedum alfredii Hance*, an HM-hyperaccumulator, transcriptome analysis showed that cell wall biosynthesis genes encoding *CesA* and *XTH* were up-regulated [255]. In addition to the above-mentioned study, several transcriptome analyses have been performed on plants exposed to HM, revealing that cell wall-related genes are altered. A large-scale rice transcriptome analysis under arsenate (As(V)) stress showed changes in gene expression including 40 cell wall-related genes among 637 transcripts [222]. Of these, 13 were up-regulated and 27 down-regulated. Down-regulated genes were cellulose synthase like A (*CslA*), xyloglucan galactosyltransferases (*XGT*), xyloglucan xylosyltransferases, galactomannan galactosyltransferases (*XXT*), glycosyl transferase (GT8, pectin synthesis), xyloglucan endotransglycosylases/hydrolases (*XTH*), β-galactosidases (βGAL), glycoside hydrolases 9 (GH9) and polygalacturonases (GH28). The down-regulation of these genes could limit cell expansion, as root growth is inhibited under arsenic stress. In *Medicago truncatula*, cell wall-modifying genes, including pectin esterase and pectin esterase precursors, were up-regulated following treatment with Al [250]. Furthermore, microarray analysis performed on rice exposed to metal stress showed that WAK proteins were up-regulated in rice subjected to As(V) stress, indicating that WAK may be involved in heavy metal stress tolerance [222]. The presence of heavy metals in the cell wall does not always result in cell wall stiffening and inhibition of cell elongation. For example, the presence of Cu and Fe can lead to polysaccharide scission, promoting cell elongation. Pectin and xyloglucan can be broken down by hydroxyl radical (^•^OH) production generated by hydrogen peroxide in the presence of ascorbate and a transient metal ion [256]. Thus, (^•^OH) free radicals produced in the cell wall under metal stress could act as a site-specific oxidant, targeted to play a role in the cell wall loosening process supporting cell expansion [257].

Lignin biosynthesis is induced under HM exposure. Cu had a positive effect on the biosynthesis of lignin and increased activities of several enzymes, including PRX and laccases, which carry out the polymerization of monolignol precursors of lignin [258]. In addition, in *Panax ginseng* exposed to a higher level of Cu, PAL and CAD enzyme activities were induced. This was correlated with an accumulation of lignin, indicating a protective response to higher levels of Cu [259]. Furthermore, transcriptome analysis of rice seedling revealed that the expression of 6 lignin biosynthesis genes was increased in rice roots under As(V) exposure. A previous study indicated that lignification might be an important step in root growth reduction in plants exposed to heavy metal stress. Therefore, As(V) may have a significant effect on the inhibition of root elongation by regulating cell wall organization. The ROS production induced by As(V) exposure may contribute to cell wall rigidity and inhibition of rice root growth. A similar trend has been observed for *Medicago truncatula* exposed to Al stress, where genes encoding *4CL* and *COMT* were up-regulated in response to Al stress in root tips [250]. It was proposed that the extent of root growth inhibition in response to Al was closely correlated with the extent of lignin deposition. It is conceivable that the Al-induced increase in activity of peroxidases triggered lignin production resulting in cell wall stiffening and reduced root growth in *M. truncatula* [250]. In a Cd-hyperaccumulator plant, *Sedum alfredii* *Hance*, genes encoding laccase and *C4H* were not induced but expressed at a higher basal level compared with the non-hyperaccumulating genotype [255].

**Figure 6:**
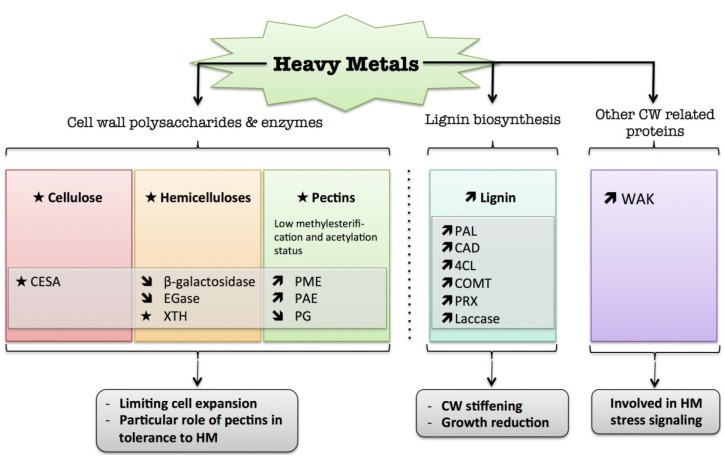
Diagram summarizing the plant cell wall response to heavy metals. The schematic presentation is deduced from the results of different studies reported in the review. Arrow (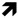) means increased abundance and arrow (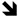) means decreased abundance of the molecules. Star (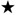) means contrasting data on the gene/protein or the molecule studied according to the literature cited in the review. Cell wall (CW); xyloglucan endo-β-transglucosylases/hydrolases (XET/XTH); endoglucanase (EGase); pectin methylesterase (PME); pectin acetylesterase (PAE); polygalacturonase (PG); phenylalanine ammonia-lyase (PAL); hydroxycinnamyl alcohol dehydrogenase (CAD); 4 coumarate CoA-ligase (4CL); caffeate *O*-methyltransferase (COMT); cell wall peroxidases (PRX).

Taken together, these data show a key role for cell wall composition particularly that of pectins, in the tolerance of plants to heavy metals, and could open new perspectives to engineer HM-resistant genotypes (Figure 6).

## 8. Light

Although light, which contains Ultra Violet (UV), visible and far-red wavelengths, is essential for plants to ensure normal growth, its excess can cause cellular damage. Plants have therefore developed means of protection, notably through changes in the cell wall.

### 8.1. Shade Avoidance

Plant-to-plant competition is an example of environment sensing and response. A shade avoidance system (SAS) is present in several plant species upon the detection of shade signals from neighboring plants, notably within dense populations [260]. Following sensing through the absorption of light by photoreceptors, it enables morphological responses and differential growth to reach the upper canopy [261]. A reduction in blue and red light (red to far-red ratio) is an important factor that is sensed by the plant photoreceptors that trigger stem and petiole elongation, primarily through cellular expansion [260]. This elongation is a consequence of cell wall loosening mediated by cell wall-modifying enzymes such as expansins and XTHs [262].

A microarray analysis performed on Arabidopsis seedlings identified two *EXP* genes (N37536 and R29778) whose expression was modified by changes in light quality [263]. Similarly, in *Stellaria longipes*, five expansin genes, among the 12 expansin sequences identified from the internodes, were up-regulated relative to the controls in response to changes in light regimes [264]. The key role of the fine-tuning of the cellulose-hemicellulose network in response to light can be further illustrated by the regulation of *XTH*. In Arabidopsis, the expression of five *XTH* genes (*XTH9*, *-15*, *-16*, *-17*, and *-19*) out of 33 was increased by low red to far-red light conditions. In parallel, the expression of *XTH16*, *-17*, *-19* and *-22* was significantly increased in response to green shade which mimics white light conditions in a dense canopy (combined reductions of blue, R/FR, and total light intensity). The functional role of XTH in the shade response was shown by characterizing knockout mutants for two of these *XTH* genes (*XTH15* and *XTH17*), which revealed reduced or absent shade avoidance responses to these light signals. This ultimately demonstrated that cell wall loosening is a key element in the plant response to shade avoidance [265].

### 8.2. Light Irradiance

As observed in pea and lettuce, light is involved in the regulation of cell wall sugar content in plants, which depends on the tissue or organ considered. [266,267]. In etiolated pea seedling, stem elongation is inhibited by about 75% following both blue and red light exposure as compared to dark controls. The fast inhibitory response was mediated by blue light while the long one was induced by red light. The degree of growth rate inhibition was increased by longer exposure to red light [268]. That inhibition was due to changes of viscoelastic properties of the cell wall.

During Bartlett pear (*Pyrus communis* L.) ripening, cell wall catabolism occurred in both sun- and shade-grown fruits, but pectin solubilization was clearly delayed in sun-exposed fruit. This was associated with a decreased removal of RG I-arabinan side chains rather than reduced depolymerization of the entire polymer. After ripening, sun-exposed pears remained firmer, and there was not a great impact on pectin molecular weight. At harvest, however, a higher proportion of water-solubilized uronic acids and alkali-solubilized neutral sugars and a larger mean molecular size of tightly bound glucans were found in sun-exposed pears [269]. Light radiation altered genes encoding proteins involved in cell wall modifications [155]. The overexpression of a blue light photoreceptor, *BnCRY1*, in *Brassica napus* reduced seedling elongation. In parallel, the expression of cell wall related genes including *XTH* and *PME* showed a 2.5- and 4-fold decreased level respectively in that transgenic line [270].

The lignin biosynthetic pathway is regulated by the photoperiod and light quality [24]. Changes in light conditions have been shown to be associated with an increase in lignin content in a number of different developmental processes and species. *Ebenus cretica* L. seedlings grown in the light displayed 2.5 times more lignin than those grown in the dark [271]. The observed alteration in leaf morphology in response to changing light conditions could thus be the consequence of modifications of the structural components of lignin. Similar results were observed in coffee leaves exposed to sunlight, where the lignin content in leaves has been positively linked to radiation, with an increase in the bulk elasticity modulus that could be explained by the higher content of lignin when compared with shade leaves [24]. Light intensity has also been shown to exert an effect on the activity of enzymes involved in the lignin biosynthetic pathway. Analyses of orchid plants exposed to different light intensities, after transfer from *in vitro* to soil, revealed that PAL and CAD enzyme activities were induced in leaves [272]. In Arabidopsis, 7000 genes exhibited altered expression when plants were subjected to high light intensity. Among them, 110 showed an increased expression, and several of them, including *4CL*, are involved in the lignin biosynthetic pathway [273].

### 8.3. UV Light

In recent years, there has been an increasing interest in the biological effects of UV light (230–400 nm) on plants. UV-B radiation (280–315 nm) reduced leaf elongation in Antarctic grass (*Deschampsia antarctica*) and in lettuce, when compared with plants exposed to reduced UV-B. This could be partly explained by the accumulation of insoluble hydroxycinnamic acids (*p*-coumaric, caffeic and ferulic acids) cross-linking the cell wall polysaccharide network and cell wall PRX thus limiting the expansion of epidermal cells and leaf elongation [267,274]. Among the effects of UV-B, cell wall elasticity was reduced in grapevine cells [275]. In cell wall isolates from pea leaves or on citrus fruit pectin, UV-A (315–400 nm) or UV-B produced hydroxyl radical (^•^OH) in presence of hydrogen peroxide. Pectin is able to transform (^•^OH) to superoxide (O_2_^•−^), which may have an intracellular impact, since it can pass cellular membrane [276]. (^•^OH) released could contribute to methane production from galacturonic acid methyl ester groups, and also, at a lower level, from acetate groups, [276,277]. This indicates that pectin might be involved in the UV radiation signaling pathway as a source of superoxide [276,278]. In tomato pericarp, UV-C irradiation (230–280 nm) exposure during postharvest retarded cell wall disassembly. This was notably related to the down-regulation of transcriptional expression or through the inhibition of the activities of many cell wall-degrading enzymes, including PME, PG and cellulase, during ripening [279,280]. 

Changes in cell wall structure and composition under UV light could produce mechanical barriers and protective substances such as secondary compounds including lignin [281]. In cucumber, cotyledons exposed to UV-B radiation lignin accumulated in cell walls of the parenchyma of tracheid elements and in the intercellular space [282,283]. Phenylpropanoids, such as hydroxycinnamic acids and their derivatives (sinapate esters), as well as flavonoids, might play complementary roles in UV-B protection. In addition, in grapevine leaves, the expression of genes of the lignin biosynthetic pathway, such as *PAL*, *C4H*, *4CL* and *CCoAOMT*, were all up-regulated in response to UV-B radiation while genes associated with cell wall loosening were down-regulated [284,285].

**Figure 7:**
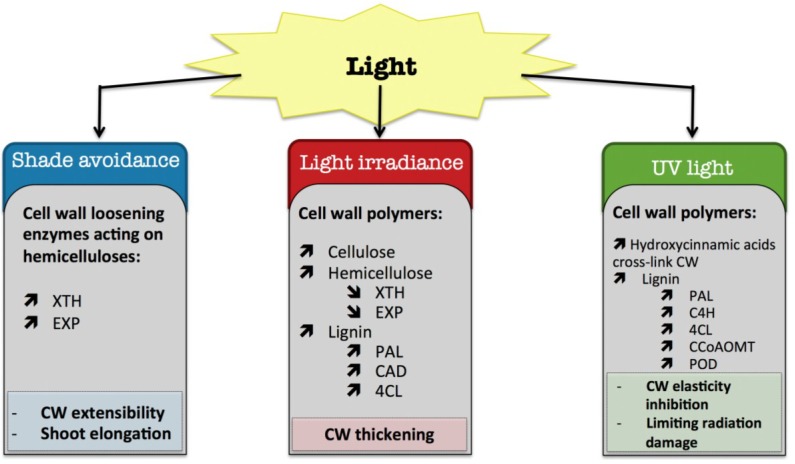
Diagram summarizing the plant cell wall response to light. The schematic presentation is deduced from the results of different studies reported in the review. Arrow (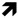) means increased abundance and arrow (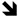) means decreased abundance of the molecules. Star (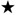) means contrasting data on the gene/protein or the molecule studied according to the literature cited in the review. Cell wall (CW); xyloglucan endo-β-transglucosylases/hydrolases (XET/XTH); expansin (EXP); phenylalanine ammonia-lyase (PAL); hydroxycinnamyl alcohol dehydrogenase (CAD); 4 coumarate CoA-ligase (4CL); caffeoyl-CoA 3-*O*-methyl-transferase (CCoAOMT); trans-cinnamate 4-hydroxylase (C4H); cell wall peroxidases (PRX).

These rather scarce data show that light intensity and quality influence cell wall architecture, particularly lignin, which could attenuate radiation and thus protect plant tissues and organs. However, the mechanism of cell wall regulation by light remains to be investigated. An overview of the plant cell wall response to light is presented in Figure 7.

## 9. Air Pollutants

According to the US environment protection Agency (EPA) the main air pollutants are ozone, particle, carbon monoxide, nitrogen oxide, sulfur dioxide and lead. Among these factors the main pollutants affecting ecosystems are carbon dioxide (CO_2_) and ozone (O_3_) [286]. CO_2_ is the primary greenhouse gas emitted through human activities (U.S. environment protection Agency, EPA) and is the substrate for photosynthesis. Its increased concentration in the atmosphere can stimulate plant growth photosynthesis and the accumulation of plant biomass in the short-term. But, elevated atmospheric CO_2_ is accompanied by a decrease in oxygen levels which thus can reduce photorespiration, a major source of hydrogen peroxide. This may then reduce abiotic stress response signaling pathway [287]. The tropospheric ozone concentrations (O_3_) are more than double compared to the levels measured over a century ago [288]. (O_3_) are high enough to cause damage to crops and thus decrease crop yields [289]. Unlike CO_2_, O_3_ is rapidly converted to ROS. It then interferes with basic physiological functions by accelerating foliar damage, senescence and reducing photosynthesis [290]. This chapter only reports data on the effects of ozone exposure and elevated atmospheric CO_2_ on cell wall metabolism since these two main air pollutants for which a number of data is available.

### 9.1. Ozone (O_3_)

Ozone (O_3_) is considered the most important consequence of air pollution and is harmful to plants, animals and humans [291]. The cell wall has been shown to be an early target of ozone [292]. The plant response to ozone is thus dependent on the species, the age of the plant, the season, the time of day and, ultimately, ozone concentration [293,294]. For instance, tobacco was more sensitive to O_3_ than deciduous trees while juvenile Manna ash (*Fraxinus ornus* L.) trees were more sensitive than mature trees [295]. O_3_ induces mainly leaf and root injuries, which can lead to biomass reduction, as observed in tobacco, poplar and birch [291,295,296]. In Manna ash (*Fraxinus ornus* L.), one of the main structural impacts of O_3_ was punctures within the cell wall, as observed on leaf mesophyll cells [295]. The development of symptoms was accompanied by the thickening of Manna ash (*Fraxinus ornus* L.) palisade mesophyll cell walls, which appears to be a common feature in several species [295,297]. Cell wall thickening is accompanied by an accumulation of cellulose layers in woody plant species including, *Fagus sylvatica*, *Acer pseudoplatanus*, which reduces the inner space of the cells [297,298]. Cell wall thickening facilitates detoxification processes and confers greater mechanical resistance to cell collapse [297,299]. In Manna ash, pectins and other cell wall polysaccharides are likely to be key components of this cell wall thickening, as shown by the cytochemical cell wall composition of stomata, which displayed more cellulose and lignin in the outer cell wall layer and more pectin in the inner cell wall layer [293,295]. This might contribute to maintaining the mechanical properties of these cells [295]. In ragweed (*Ambrosia artemisiifolia* L.) pollen, FTIR analysis showed a decrease in phenolic compounds and an increase in pectin components (rhamnogalacturonic acid, arabinan) under elevated ozone [300]. Notably, a decrease in acetylester groups, indicating a de-esterification of pectin in response to ozone, was shown. Transcriptome analysis further revealed that the expression of pectin methylesterase and pectate lyase encoding genes was strongly induced in ragweed pollen in response to ozone. Although Amb a1, an allergen found in *Ambrosia artemisiifolia* pollen, which is a pectate lyase, was highly expressed in response to ozone, no significant difference was measured at the protein level, as tested by an ELISA experiment [300]. Cell wall sugar content increased under ozone exposure in strawberry plant, which was related to increased pectin content (100% more) after a short treatment (7 days) rather than long exposure (2 months). These changes were primarily due to water-soluble pectin suggesting that either pectin degradation was decreased or *de novo* synthesis increased. In parallel, long-term exposure reduced non-soluble pectin by 60% and 80% in more and less O_3_-tolerant strawberry cultivars, respectively. This appeared to be related to changes in the amount of low methoxy pectin content, which increased in the tolerant genotype and decreased in the sensitive one.

O_3_ can be used to delay fruit ripening during cold storage, due to its ability to inhibit ethylene oxidizing capacity. In kiwifruit, O_3_ treatment at 0 °C reduced cell wall disassembly of the fruits during ripening at room temperature [301]. In that study, the swelling capacity of the cell wall associated with the solubilization of pectin was reduced when fruit was treated with O_3_. The treatment induced lower PG and endo-1,4-β-glucanase activities, enzymes involved in pectin and hemicellulose degradation, compared with the control environment. In contrast to the above-mentioned findings, in O_3_-sensitive aspen, leaves were thinner compared to the tolerant genotype, concomitant with a reduction in cell walls. The average thinner cell walls in mesophyll cells in the sensitive aspen clone could decrease cell protection from O_3_ present in the apoplast [302]. At the transcriptional level, in an O_3_-sensitive rice cultivar, genes encoding cell wall degradation enzymes, including cellulase and β-galactosidase, were up-regulated. Other genes encoding cell wall-related proteins, such as cell wall invertase inhibitor or glycine-rich protein, were similarly up-regulated while the expression of genes encoding proteins involved in cell wall biosynthesis, such as *XET* and *COBRA*, was down-regulated [303]. In contrast, in the O_3_-tolerant rice genotype, a different transcriptional regulation is likely to occur, with an increased expression of genes encoding some expansin and xylanase inhibitors.

O_3_ also has an impact on wood formation. In poplar, stems were more affected than leaves, with a reduction in stem diameter and the anatomy of the tension wood [296]. O_3_ reduced cambial growth and xylem differentiation, which was associated with a reduction in cellulose and lignin biosynthesis [296]. This was accompanied by changes in the expression of specific genes, and by a decrease in activity of enzymes involved in cellulose (SuSy, UGPase) and lignin formation (PAL, CAD) [296]. In poplar exposed to ozone, changes in lignin structure occurred, with enrichment in condensed lignin by a higher proportion of H-units near the foliar necrotic area [304]. CAD activity was rapidly and strongly increased (10-fold) whatever the foliar developmental stage, while PAL enzyme activity was increased in old and mid-aged leaves [304]. In non-woody plants, at the transcriptome level, *PAL* mRNA, a key enzyme involved in the phenylpropanoid pathway including lignin biosynthesis, was rapidly and transiently induced reaching a 3-fold higher level compared to control Arabidopsis or parsley plants [305,306]. In contrast, O_3_ was also found to modify cell wall components by depolymerization of lignin, which released small phenolic compounds that could be used as elicitor molecules [307].

**Figure 8:**
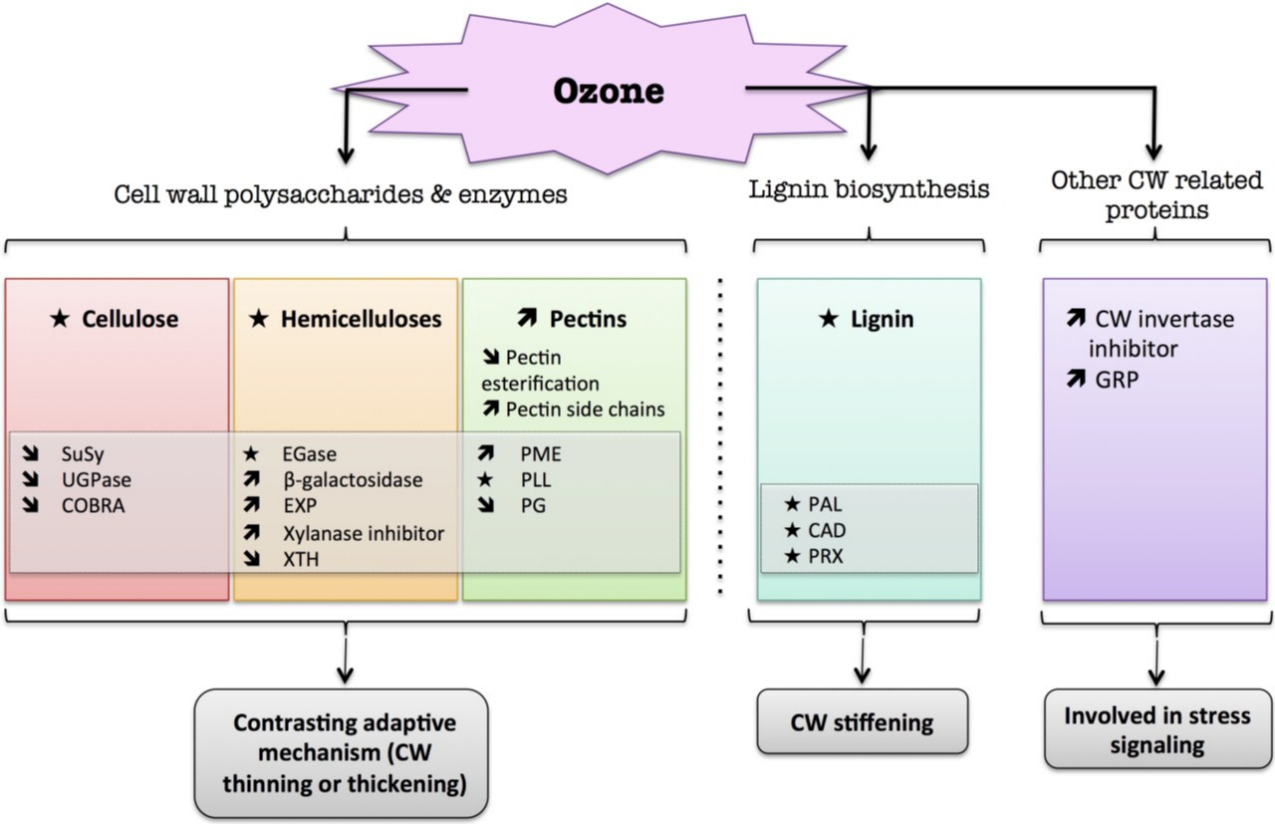
Diagram summarizing the plant cell wall response to ozone. The schematic presentation is deduced from the results of different studies reported in the review. Arrow (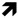) means increased abundance and arrow (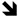) means decreased abundance of the molecules. Star (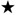) means contrasting data on the gene/protein or the molecule studied according to the literature cited in the review. Cell wall (CW); xyloglucan endo-β-transglucosylases/hydrolases (XET/XTH); expansin (EXP); sucrose synthase (SuSy); UDP-glucose pyrophosphorylase (UGPase); endoglucanase (EGase); pectin methylesterase (PME); pectin/pectate lyase-like (PLL); polygalacturonase (PG); phenylalanine ammonia-lyase (PAL); hydroxycinnamyl alcohol dehydrogenase (CAD); glycine-rich protein (GRP); cell wall peroxidases (PRX).

Cell wall peroxidases might regulate the level of ROS by scavenging H_2_O_2_ to polymerize lignin and cross-link cell wall proteins and polysaccharides [308]. In sunflower leaves exposed to ozone, cell wall PRX was induced thus leading to cell wall stiffening and contributing to slowing down O_3_ penetration into the cell [308,309]. Furthermore, PRX displayed higher activity with syringyl compared with guaiacyl molecules. In addition, PRX activity in strawberry leaves decreased in the less tolerant cultivar while it increased in the ozone-tolerant genotype under short term ozone exposure, indicating a significant contribution of cell wall PRX to the detoxification of ozone in the apoplast [310] These data, which are mostly derived from cytochemical analysis and enzyme activity measurements, implies a contribution of cell wall proteins to apoplastic ozone detoxification. In addition, some genomic approaches provided valuable knowledge about the role of the plant cell wall in response to ozone (Figure 8). However, very few data are currently available at the biochemical level on cell wall polymers, cell wall enzyme activities and signaling processes that regulate this response.

### 9.2. CO_2_

The atmospheric CO_2_ level has increased by 30% over the last 250 years and is forecast to rise by 1%–2% per year [311]. This has an impact on plant development as C3 crops show a significant increase in photosynthesis and growth in response to rising CO_2_ while C4 plants, including maize, Miscanthus, and sugarcane differ in their response [312,313].

In woody plants, including poplar, growing under elevated CO_2_, trees have a larger leaf size, increased stem and branch growth and root biomass [314,315,316,317,318,319,320]. CO_2_ responsiveness was found to be dependent on genotype. The unresponsive aspen clone grew slower than the responsive one, suggesting different strategies used in carbon partitioning [317]. A significant decrease in cell wall thickness was observed in the CO_2_-responsive clone [317]. During the late season, the CO_2-_unresponsive clone displayed a cell wall thickening in the leaves, which is likely to be related to an increased level of UDP-sugar pyrophosphorylase transcripts. In contrast, a long term exposure (11 years) to an elevated CO_2_, decreased cell wall thickness in different aspen genotypes and in sweetgum (*Liquidambar styraciflua* L.), suggesting that a short term exposure may not provide a realistic view of a long term exposure [319,320]. In addition, no significant changes in the cell wall sugar content were observed [320]. In herbaceous plants, including Arabidopsis, grown under elevated CO_2_ conditions, a rise in whole plant biomass (+20%) was observed before flowering. UDP-Glc dehydrogenase, a key sugar in the nucleotide interconversion pathway, necessary for cell wall polysaccharide biosynthesis, was increased in these young plants. However, although the CO_2_ concentration increased the dry weight of the cell wall, it had no significant effect on cell wall composition as observed in sweetgum [320,321]. In Arabidopsis, an increase in plasmodesmata formation in the midrib of the first leaves exposed to elevated CO_2_ was observed. This might be related to a higher sucrose translocation in the sieve elements [322]. In barley, under ambient CO_2_, cell wall elasticity was decreased compared to elevated CO_2_ levels, which could be considered as a positive mechanism for maintaining cell turgor and preventing cell wall rupture [323]. In addition, high CO_2_ was shown to have an effect on post-harvest biochemical and textural properties of white asparagus spears, based on changes in cell wall components [324]. For instance, during post-harvest storage, high CO_2_ levels inhibited the excessive accumulation of secondary wall components, including cellulose and lignin, at room temperature (20 °C), thus decreasing the toughening of asparagus spears. Pectin content was less influenced by elevated CO_2_ compared to secondary cell wall components. In contrast, at lower temperature (10 °C) and elevated CO_2_ atmosphere, no significant changes in cell wall composition and distribution were observed compared with cold applied on its own. Thickening of cell walls and lignification could be delayed by elevated CO_2_ at room temperature. This could explain why elevated CO_2_ partially inhibited PAL activity, as observed in green asparagus grown in an oxygen modified atmosphere [325]. In arrowhead, shoot elongation was stimulated by elevated CO_2_. This growth phenotype was associated with higher transcript levels of genes encoding *EXP* and *XTH* genes, both of which are known to control cell wall loosening during cell growth [326]. MicroRNAs which are important regulators of gene expression are altered under long-term elevated CO_2_ in Arabidopsis. For instance, miRPAL2 which regulates, cellulose synthase genes including *AtCesA10*, pectin biosynthesis gene including galacturonosyltransferase-like 9 or glucosyltransferase family 8 (GT8) is up-regulated, indicating an alteration of plant cell wall biosynthesis under elevated CO_2_ [327].

Another, transcriptome analysis performed on old aspen trees after 12 years of exposure to elevated CO_2_, showed a significantly up-regulatation of genes encoding enzymes involved in cell wall loosening and expansion (*XTH*, *EXP*). This indicates a radial growth expansion in response to elevated CO_2_ [318]. In addition, other genes involved in cellulose biosynthesis, non-cellulosic and pectin biosynthesis had increased transcript levels. The overall average expression of these genes was 33% higher in the vascular cambium zone (VCZ), which reflects a more intensive cell wall biosynthesis there. Pectin-remodeling enzymes, including pectate lyase, pectin acetylesterase and pectin methylesterase, were highly up-regulated in response to elevated CO_2_, and a *PME* orthologous to *AtPME3* was up-regulated by at least 25% in the VCZ. Similar patterns of expression were shown for a putative pectin acetylesterase gene and a number of NDP-sugar epimerases (UDP-glucuronate 4-epimerase, UDP-glucose 4-epimerase, and UDP-arabinose 4-epimerase (MUR4)). Overall, this indicates a significant increase in pectin and hemicellulose biosynthesis in vascular meristematic cells in response to growth under elevated CO_2_. In contrast, genes involved in lignin biosynthesis were mostly down-regulated in the VCZ (24%) and in leaves (11.4%) indicating that down-regulation of lignin biosynthesis is important for maintaining cell growth in this zone [318]. In another atudy, performed on Aspen leaves of both unresponsive and CO_2_ responsive genotypes revealed a distinct response between them during both early and late growing seasons. During the early season, the expression of genes encoding cell wall glycosyltransferase (GT43) was increased in both clones in elevated CO_2_ conditions. In contrast, some other genes encoding cell wall-remodeling enzymes were differentially regulated. For instance, genes encoding enzymes involved in secondary cell wall metabolism, including cell wall *PRX*, were up-regulated in the CO_2_-unresponsive clone. Furthermore, a strong up-regulation of NAC protein (for NAM, no apical meristem), a transcription factor known to regulate secondary cell wall synthesis genes in fibers, was observed in the sensitive clone leaves. This would suggest that NAC could control the carbon deposition in the leaf secondary cell wall thickening of the sensitive clone in response to elevated CO_2_ [317]. In contrast, in the genotype that displayed a high responsiveness to elevated CO_2_, genes encoding enzymes involved in lignin biosynthesis, including *CCoAOMT* and laccase, were down-regulated in leaves during the early growing season. No significant changes were observed in the late growing season in that genotype. A similar trend was observed for fascilin-like arabinogalactan protein and pectin methylesterase genes. The responsive clone is likely to have a strategy that maintains the ability to grow while the sensitive one devotes more energy to passive defense, *i.e.*, lignin biosynthesis and cell wall thickening.

**Figure 9:**
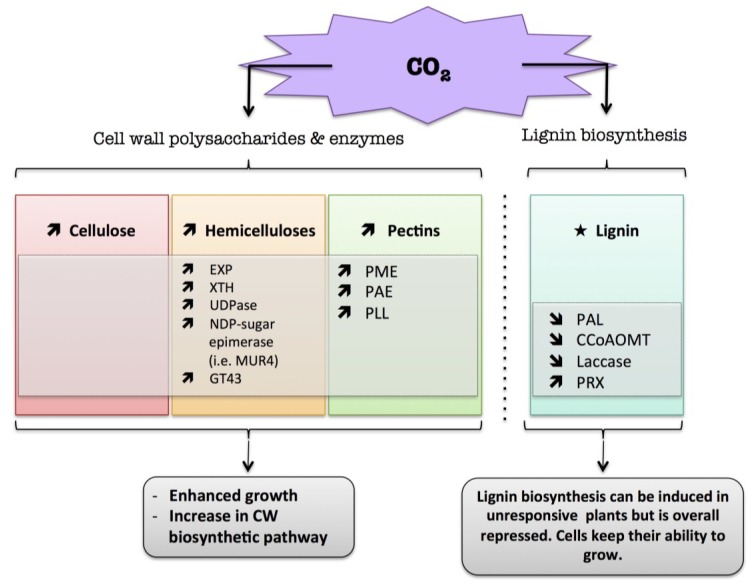
Diagram summarizing the plant cell wall response to elevated CO_2_. The schematic presentation is deduced from the results of different studies reported in the review. Arrow (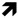) means increased abundance and arrow (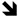) means decreased abundance of the molecules. Star (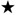) means contrasting data on the gene/protein or the molecule studied according to the literature cited in the review. Cell wall (CW); xyloglucan endo-β-transglucosylases/hydrolases (XET/XTH); expansin (EXP); UDP-glucose pyrophosphorylase (UGPase); pectin methylesterase (PME); pectin acetylesterase (PAE); pectin/pectate lyase-like (PLL); glycosyltransferase family 43 (GT43); phenylalanine ammonia-lyase (PAL); caffeoyl-CoA 3-*O*-methyl-transferase (CCoAOMT); cell wall peroxidases (PRX).

All these data confirm that atmospheric pollution has an impact on plant cell wall metabolism (Figure 8 and Figure 9). However, these data are scarce and studies need to be developed to supply plants adapted to such environments in the future.

## 10. Conclusions

**Figure 10:**
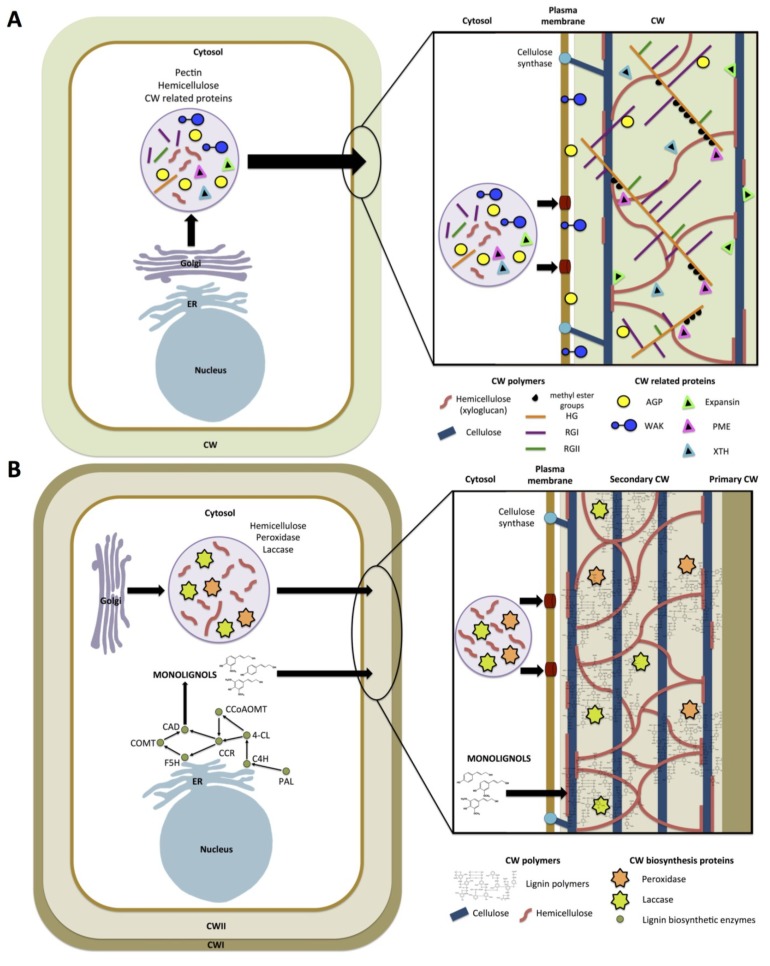
Model depicting changes in the plant cell wall mechanism in response to abiotic stress. (**A**) Key points of the dynamics of type I primary cell wall (CW) during abiotic stress exposure are shown. Cell wall polymer biosynthesis takes place at different locations within the cell. During cell wall formation, hemicelluloses and pectin are synthesized in the Golgi apparatus of the plant cell and then secreted to the apoplastic space while cellulose synthesis occurs at the plasma membrane. The organization and interactions of wall components is coordinated with dynamic assembly in the apoplast and rearrangement occurs during wall extension [328]. During growth under non-optimal environmental conditions, the primary cell wall composition is altered. In several abiotic stresses, cellulose, hemicellulose and pectin contents are changed. Different adaptive mechanisms can be observed, which depend on the species considered, the organ, the tissue, the age, and the exposure intensity of the stress. In most “tolerant” species, the biosynthesis of cellulose and xyloglucan, which is the most abundant non-cellulosic component of type I primary walls, is induced. This is associated with an up-regulation of the expression of genes encoding *EXP* (expansin) and *XTH* (xyloglucan endo-β-transglucosylases/hydrolases). In parallel, levels of rhamnogalacturonan I (RGI) enriched in arabinan and/or galactan side chains are increased. The level of methylesterification of homogalacturonan (HG), regulated by PME (pectin methylesterase), is reduced. All these modifications of the cell wall architecture lead to a relative maintenance of cell wall extensibility, adapted to coping with a particular abiotic stress. For species that are “sensitive” to a given abiotic stress, cell wall degradation is observed, related to a decrease in cell wall polysaccharide content, an increase in cell wall hydrolases (EGase) and a decrease in cell wall biosynthesis and remodeling enzymes (PME, PG, SuSy). Abiotic stress can also alter wall-associated kinases (WAK), which are required for cell elongation and development. In plants that display tolerance to abiotic stress, the expression of genes encoding *WAK* are up-regulated, which suggests a perception of the stress at the cell wall/plasma membrane interface through the detection of released plant cell wall fragments [187,188,222]. Genes encoding other cell wall proteins, including arabinogalactan protein (AGP), proline-rich protein (PRP) and glycine-rich protein (GRP) are induced in response to abiotic stress, which could covalently link with pectin or hemicellulose thus contributing to the strengthening of the wall [329]. Another possibility is that HRGP is a signal molecule in plant defense [330]; (**B**) Key points of the secondary cell wall (CWII) during abiotic stress exposure are shown. The model is adapted from [78]. To cope with abiotic stress, some plants adopt a strategy that consists of increasing the secondary cell wall thickening by hemicellulose and cellulose synthesis [147,295]. This is often associated with a rigidification of the secondary wall by lignin deposition [23,182,282]. Monolignols, which are the building blocks of lignin, are synthesized from phenylalanine through the general phenylpropanoid and monolignol-specific pathways. This involves cytosolic enzymes including phenylalanine ammonia-lyase (PAL), 4 coumarate CoA-ligase (4CL), cinnamoyl-CoA reductase (CCR), caffeate *O*-methyltransferase (COMT), and caffeoyl-CoA 3-*O*-methyl-transferase (CCoAOMT) as well as ER membrane-anchored proteins including trans-cinnamate 4-hydroxylase (C4H), and ferulate 5-hydroxylase (F5H). The monolignols are then transported to the cell wall where they are polymerized by apoplastic peroxidase (PRX) and laccases into lignin [117,144]. This other adaptive mechanism, based on a decrease in cell wall expansion and cell extensibility, can limit water loss and prevent cell collapse due to dehydration.

The present review outlined the impact of various abiotic stresses on the plant cell wall. Overall, cell wall architecture is affected by abiotic stress, but the extent of the available data differs depending on the stress considered. Although changes in the cell wall in response to drought or cold have been quite well documented, data concerning some other stresses, including flooding and air pollutants, are scarcer. Furthermore, the data available in the literature are more related to transcriptome and proteome analysis than to the detailed chemical composition of the cell wall in response to stress. Stress exposure, depending on the species and stress intensity, appears to result in both plant cell wall loosening and tightening. One possible strategy is thus to improve the viscoelastic properties of the primary wall by increasing levels of cell wall remodeling and biosynthesis enzymes, and by modulating other wall loosening agents, including pectin, thus contributing to increasing the hydration status of the plant and maintaining turgor pressure for growth (Figure 10A). This could enable a relative growth status to be maintained. Another option is to modulate the viscoelastic properties by reinforcing the secondary wall with cellulose deposition and non-cellulosic components. Tightening which corresponds to a decrease in cell wall extensibility and/or turgor pressure is often accompanied by lignin biosynthesis in response to stress, except during flooding, when genes and/or enzymes involved in lignin biosynthesis are repressed/inhibited (Figure 10B). However, as the review reports, the stress effects on plant cell wall architecture depend on the plant species, plant genotype, and the age of the plant, as shown under ozone exposure or in response to cold. It also relies on the timing and intensity of the stress, as shown under flooding. Clearly, cell wall adjustment under abiotic stress is an important phenomenon in plant adaptation.

Although much progress has been made over the last decade in understanding the consequences of abiotic stress on cell wall metabolism, a multidisciplinary approach including physiology, biochemistry, proteomics and genetics based on one species for each given stress could provide more consistent results to elucidate the complex mechanism of the cell wall in its response to abiotic stress. Research needs to be pursued to engineer plants with cell walls adapted to global warming in the future.
